# LYSG101: a potent chimeric lysin with therapeutic potential for combating *Staphylococcus aureus* infections

**DOI:** 10.1128/aac.01730-25

**Published:** 2026-04-30

**Authors:** Wen Xiao, Tian Fang, Jiawen Guan, Jie Ren, Mya Thandar, Guanghuai Jin, Menglin Wang, Ning Gan, Fuye Ma, Assaf Raz

**Affiliations:** 1 Lysigen, a Precisio Biotix Therapeutics company, Wuhan, Hubei, China; Shionogi Inc, Florham Park, New Jersey, USA

**Keywords:** antimicrobial resistance, biofilm, ClyO, MRSA, peptidoglycan hydrolase, endolysin

## Abstract

Lysins are peptidoglycan hydrolases with great promise as novel and highly differentiated biotherapeutic agents. Here, we characterize LYSG101, a chimeric lysin active against all *Staphylococcus* species, including *Staphylococcus aureus*. LYSG101 exhibits potent activity against a range of clinical isolates, including methicillin-resistant *S. aureus* (MRSA) and coagulase-negative staphylococci (CoNS). Minimum inhibitory concentrations that inhibited 50% (MIC_50_) and 90% (MIC_90_) of *S. aureus* strains tested were 1 and 2 µg/mL, respectively, in cation-adjusted Mueller-Hinton broth (CAMHB) and 0.0625 and 0.125 µg/mL, respectively, in CAMHB + 25% horse serum, with no resistant outliers. LYSG101 is bactericidal, based on minimum bactericidal concentration (MBC) values within one log_2_ dilution of MIC values, and >3-log_10_ reduction within 15 min of exposure to lysin in time-kill assays. Serial passage resistance assays demonstrated no development of resistance to LYSG101 following 100 daily passages, whereas mupirocin resistance increased up to 500-fold over the same period. LYSG101 effectively disrupted *S. aureus* and CoNS biofilms. *In vivo*, LYSG101 significantly improved survival in murine models of intraperitoneal and intravenous *S. aureus* infection at 0.25 mg/kg (*P* < 0.01 for both), and resulted in a significant reduction in lung CFU (*P* < 0.0001) in a neutropenic lung infection model. These findings support the potential of LYSG101 as a new and effective therapeutic agent for staphylococcal infections.

## INTRODUCTION

Antimicrobial resistance (AMR) poses a significant threat to human health. In 2019, an estimated 4.95 million deaths were associated with AMR, with methicillin-resistant *Staphylococcus aureus* (MRSA) as one of the six leading pathogens responsible for these fatalities (1). *S. aureus* is a major cause of skin and soft-tissue infections and is a leading cause of pneumonia, surgical site, bone and joint, and cardiovascular infections, as well as nosocomial bacteremia (2–4). In 2017, an estimated 20,000 deaths were attributed to *S. aureus* bacteremia in the United States (5). Over half of hospital-acquired *S. aureus* infections involve MRSA, which is associated with worse clinical outcomes and increased hospitalization costs (3, 6, 7). *S. aureus* frequently forms biofilms on damaged tissues and implanted medical devices, which resist antimicrobial activity and immune surveillance, presenting a significant obstacle to treatment (8). Due to the limitations of existing antibiotics, treatment options for complicated MRSA infections remain inadequate, highlighting the need for innovative therapeutic strategies.

Lysins are peptidoglycan hydrolases produced by bacteriophages during the late stage of the infection cycle to degrade the bacterial host cell wall and release progeny phages. Recombinantly produced lysins have emerged as a promising novel therapeutic modality in combating AMR. Lysins exhibit rapid and targeted bactericidal activity, synergistic effects with antibiotics, a low propensity to select for resistance, efficacy against biofilms, and effectiveness against metabolically inactive persister cells (9, 10).

To date, two lysins have entered clinical development for the treatment of *S. aureus* systemic infections. Exebacase (PlySs2, CF-301) was evaluated in a superiority trial, in addition to standard-of-care antibiotics, for patients with bacteremia and endocarditis, and has shown a 42% improvement in clinical response rate and reductions in median length of hospital stays in the prespecified MRSA subgroup (11). A subsequent phase 3 trial did not meet the prespecified criteria in an interim futility analysis and was confounded by extreme patient imbalance, precluding meaningful interpretation of the results (12). Treatment of prosthetic joint infection with exebacase in combination with debridement, antibiotics, and implant retention procedure (Lysin-DAIR) has shown enduring positive outcomes in a series of compassionate use studies (13). Tonabacase (SAL200, LSVT-1701) demonstrated a favorable safety profile in a phase 1 study (14); however, a phase 2 clinical trial (NCT03089697) was halted. Therefore, while there is clear potential for lysins in the treatment of systemic staphylococcal infection, further optimization of therapeutic lysins is needed.

This study focuses on a novel chimeric lysin, LYSG101 (ClyO), derived from a fusion of two lysins (15) and, thus, distinct from a native lysin like exebacase. We report detailed microbiologic profiling of LYSG101, demonstrating favorable antimicrobial properties that include rapid bacteriolysis of *S. aureus* and coagulase-negative staphylococci (CoNS), low minimum inhibitory concentration (MIC) and minimum bactericidal concentration (MBC) values in the tested bacterial population, eradication of pre-formed biofilms, no selection for resistance development, no *in vitro* cytotoxicity, and potent activity in three models of *in vivo* staphylococcal infection. Collectively, these findings support the further clinical development of LYSG101 as a therapeutic agent for staphylococcal infections.

## RESULTS

### LYSG101 (ClyO) has more rapid lysis kinetics compared to ClyF and exebacase

We evaluated two novel chimeric lysins, ClyO (15) and ClyF (16), as candidates for clinical development, using the clinical-stage lysin exebacase as a comparator. Exebacase (PlySs2) is a naturally occurring, unmodified lysin from *Streptococcus suis* (17), while ClyF and ClyO are both chimeric lysins that share the same binding domain with exebacase but have a catalytic domain derived from the staphylococcal lysin Ply187 (18); ClyF has a short linker region connecting the two domains, while ClyO has a longer linker region.

**Fig 1:**
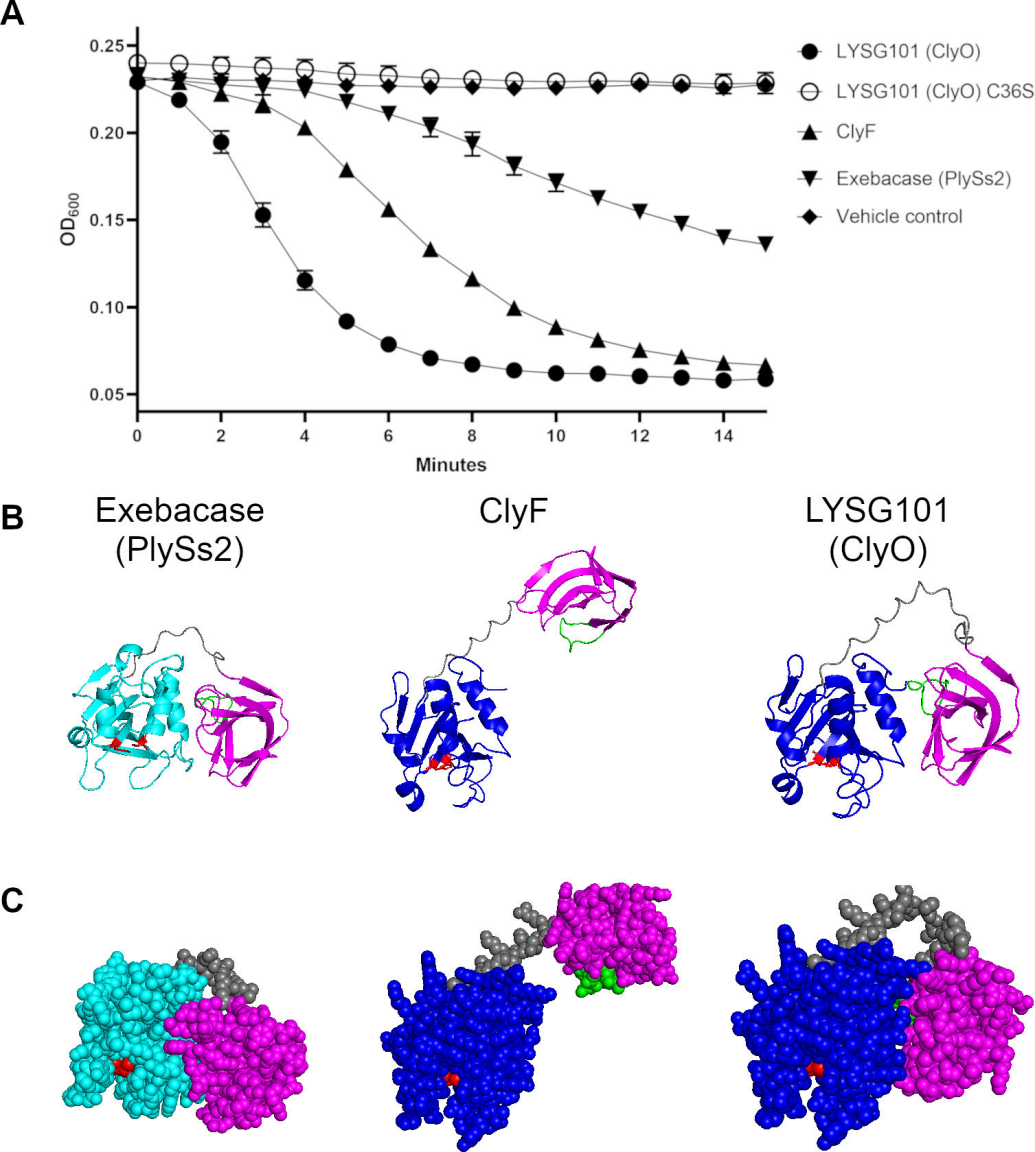
LYSG101 activity and putative structure. Lysis kinetics of *S. aureus* treated with exebacase, ClyF, LYSG101 (ClyO), and active site mutant LYSG101 (ClyO) C36S were evaluated using the OD reduction method. The lysins or vehicle control were added in triplicate in a microtiter plate to washed log-phase *S. aureus* CMCC 26003 cells at a final concentration of 0.2 µg/mL. The plate was placed in a plate reader at 37°C, and the OD_600_ was recorded every minute for 15 min (**A**); error bars represent standard deviations. Structural predictions were generated using AlphaFold2 for exebacase, ClyF, and LYSG101; structures are shown as cartoon (**B**) and sphere (**C**) representations. Colors are as follows: cyan, PlySs2 catalytic domain; blue, Ply187 catalytic domain; magenta, PlySs2 binding domain; red, active site; green, RT-loop; and gray, linker.

Lysis kinetics of *S. aureus* strain CMCC 26003 by ClyO, ClyF, and exebacase were evaluated using the optical density (OD) reduction method (19). ClyO exhibited the fastest lysis kinetics, followed by ClyF, and then exebacase (Fig. 1A). The more rapid lytic effect of the two chimeric lysins, ClyO and ClyF, may be due to the higher compatibility of their catalytic domains (of staphylococcal origin) with *S. aureus* peptidoglycan compared to the catalytic domain of exebacase (of streptococcal origin). To better understand how the difference in linker length between ClyO and ClyF might affect their catalytic activity, we performed structural predictions using AlphaFold2 (Fig. 1B and C). This analysis revealed a marked difference in domain arrangement between the two proteins. In ClyO, the catalytic and binding domains were closely associated in an orientation broadly similar to that observed in exebacase; in contrast, the catalytic and binding domains of ClyF were clearly separated. The RT loop of the binding domain (20) is highlighted in green to aid in discerning domain orientation.

Based on the substantially more rapid lysis kinetics of ClyO and its more native-like domain orientation, ClyO was selected as the preferred molecule for further development and is hereafter referred to as “LYSG101.”

### LYSG101 is a cysteine- and histidine-dependent amidohydrolase/peptidase

Domain analysis using BLASTP (21) showed the presence of a CHAP (cysteine- and histidine-dependent amidohydrolase/peptidase) superfamily catalytic domain in LYSG101, marked in part by a canonical catalytic dyad, which consists of a conserved cysteine residue at position 36 and a conserved histidine residue at position 99. To validate this, we evaluated a LYSG101 mutant, in which the catalytic cysteine residue is substituted with a serine (C36S), alongside the parental molecule. This mutant demonstrated complete abrogation of activity, thus supporting the predicted catalytic mechanism of action (Fig. 1A).

### Time-lapse microscopy evaluation of *S. aureus* lysis by LYSG101

**Fig 2:**
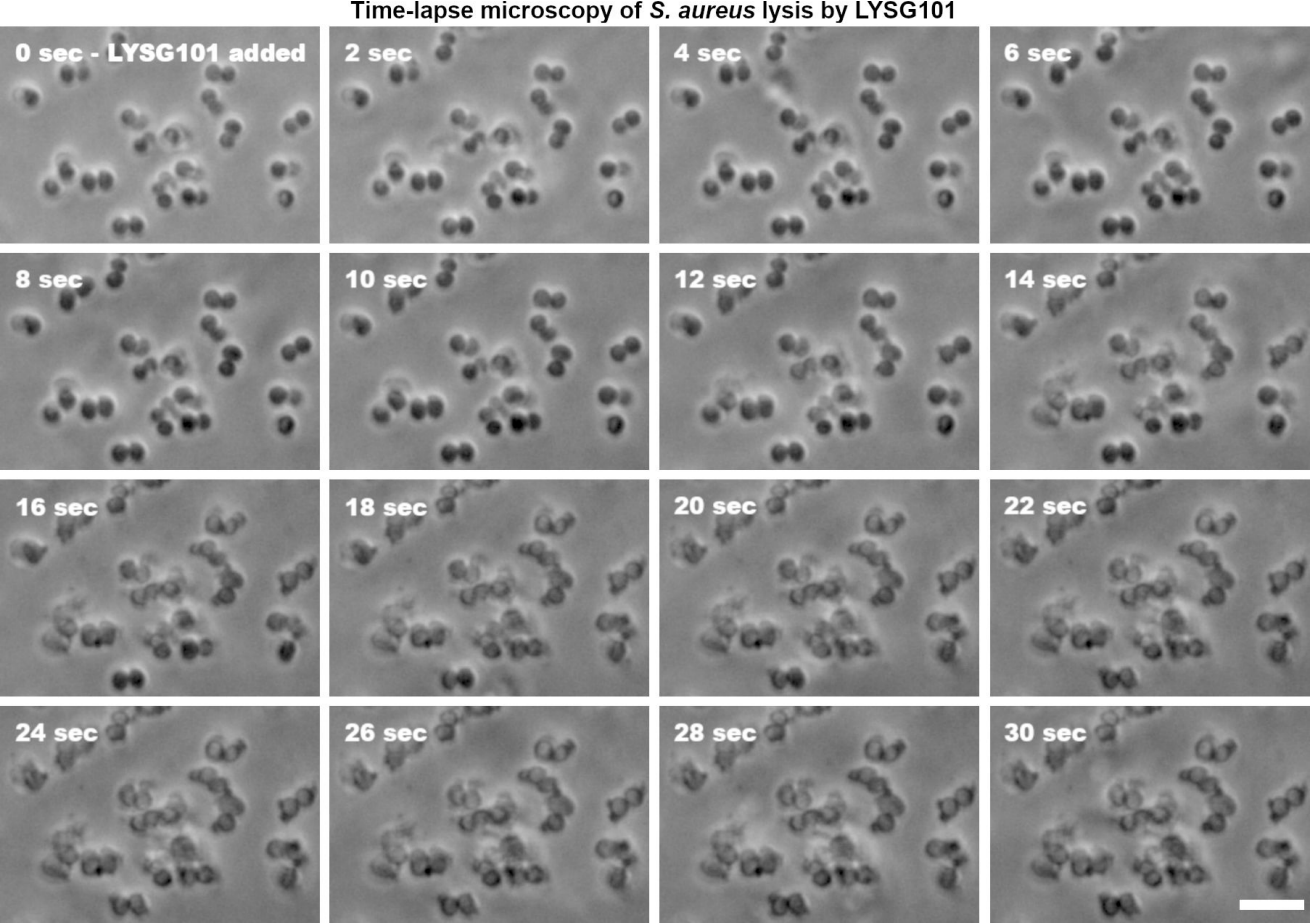
Rapid lysis of *S. aureus* by LYSG101 visualized by time-lapse microscopy. Log-phase *S. aureus* ATCC 29213 cells were washed and attached to a poly-l-lysine-coated glass-bottomed plate. The plate was washed, supplemented with DPBS, and placed on a microscope stage. DIC images were captured continuously following the addition of LYSG101. Time-lapse images of a representative field are shown. Scale bar is 5 µm; see also Video S1.

The rapid lytic activity of LYSG101 was visualized using time-lapse microscopy. Within 10 s of adding LYSG101 to a monolayer of *S. aureus* ATCC 29213, signs of cell lysis began to appear, and complete lysis was observed by 20 s (Fig. 2). Lysis was preceded by cellular swelling, after which the envelope ruptured at a single site, extruding cytoplasmic contents (Video S1). Lysis was also macroscopically evident within 30 s of adding LYSG101 to a dense staphylococcal culture (Video S2).

### LYSG101 shows favorable MICs and MBCs against a range of staphylococcal clinical isolates

**Fig 3:**
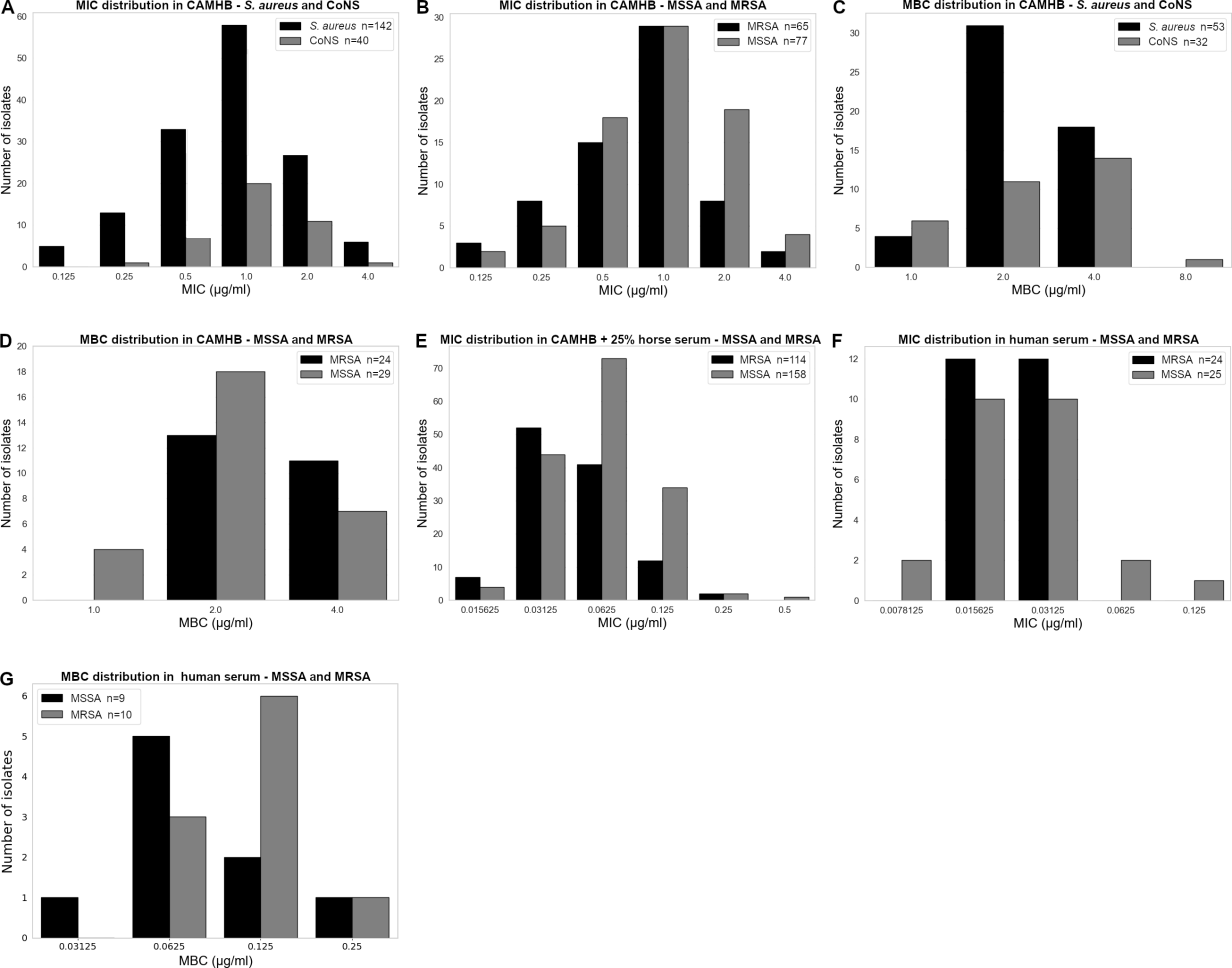
LYSG101 MIC and MBC distributions. (**A**) MIC distribution of *Staphylococcus aureus* and CoNS isolates in CAMHB. (**B**) MIC distribution of *S. aureus* isolates in CAMHB, separated into methicillin-sensitive (MSSA) and methicillin-resistant (MRSA) isolates. (**C**) MBC distribution of *S. aureus* and CoNS isolates in CAMHB. (**D**) MBC distribution of MSSA and MRSA isolates in CAMHB. (**E**) MIC distribution of MSSA and MRSA isolates in CAMHB + 25% horse serum. (**F**) MIC distribution of MSSA and MRSA isolates in human serum. (**G**) MBC distribution of MSSA and MRSA isolates in human serum.

We first evaluated the MIC distribution of LYSG101 against 142 *S. aureus* and 40 CoNS isolates in cation-adjusted Mueller-Hinton broth (CAMHB). For both *S. aureus* and CoNS strains, MIC_50/90_ values of 1/2 µg/mL were observed, with ranges of 0.125–4 µg/mL for *S. aureus* and 0.25–4 µg/mL for CoNS (Fig. 3A and Table 1). When the *S. aureus* population was further subdivided into methicillin-sensitive *S. aureus* (MSSA) and MRSA isolates, MIC_50/90_ values of 1/2 µg/mL were likewise observed, with ranges of 0.125–4 µg/mL for both (Fig. 3B). MBC_50/90_ values were evaluated for a subset of the isolates and were each 1-log_2_ dilution higher than MICs, at 2/4 µg/mL for both *S. aureus* and CoNS, consistent with a bactericidal effect (Fig. 3C). No appreciable difference in MBC values was seen between MSSA and MRSA strains (Fig. 3D).

**TABLE 1:** LYSG101 MIC and MBC distribution

	*n*	MIC_50_ or MBC_50_ (µg/mL)	MIC_90_ or MBC_90_ (µg/mL)
MIC – CAMHB			
*S. aureus*	142	1	2
MSSA	77	1	2
MRSA	65	1	2
CoNS	40	1	2
MBC – CAMHB			
*S. aureus*	53	2	4
MSSA	29	2	4
MRSA	24	2	4
CoNS	32	2	4
MIC – CAMHB + 25% horse serum			
*S. aureus*	272	0.0625	0.125
MSSA	158	0.0625	0.125
MRSA	114	0.03125	0.125
MIC – 100% human serum			
*S. aureus*	49	0.03125	0.03125
MSSA	25	0.03125	0.0625
MRSA	24	0.03125	0.03125
MBC – 100% human serum			
*S. aureus*	19	0.125	0.25
MSSA	9	0.0625	0.25
MRSA	10	0.125	0.25

Previously, serum was shown to potentiate the activity of exebacase (CF-301), driven primarily by serum albumin and lysozyme (22, 23). We evaluated the effect of human serum on the activity of LYSG101 using the checkerboard assay and observed similar potentiation of activity (Fig. S1A). We further evaluated human serum albumin (Fig. S1B) and human lysozyme (Fig. S1C) and found that both potentiated the activity of LYSG101 in a manner comparable to exebacase. Given that CAMHB + 25% horse serum may better represent *in vivo* MIC compared to CAMHB alone, a similar medium was approved by CLSI as an antibiotic susceptibility testing (AST) medium for exebacase, as described in CLSI standard M100 (24). Exebacase’s AST medium also included 0.5 mM dithiothreitol (DTT), which was needed to protect the active site cysteine in frozen panels to support large surveillance studies and clinical trials (23). In preliminary experiments, we found that inclusion of 0.5 mM DTT resulted in slightly lower MIC values compared to CAMHB + 25% horse serum without DTT (Fig. S2), as observed with exebacase (23). To establish a baseline free of additional agents, fresh panels without DTT were used in this study. For 158 MSSA isolates tested in CAMHB + 25% horse serum, MIC_50/90_ values of 0.0625/0.125 µg/mL were observed, and for 114 MRSA isolates tested, MIC_50/90_ values of 0.03125/0.125 µg/mL were observed (Fig. 3E). These values were more than 10-fold lower than MIC values in CAMHB, suggesting that like exebacase, the activity of LYSG101 was potentiated by serum components. We further evaluated MIC values for a subset of the isolates in human serum; 25 MSSA isolates showed MIC_50/90_ values of 0.03125/0.0625 µg/mL and 24 MRSA isolates showed MIC_50/90_ values of 0.03125/0.03125 µg/mL, consistent with potent activity in serum (Fig. 3F). MBC_50/90_ values were evaluated in human serum for a subset of the isolates and were 0.0625/0.25 µg/mL for MSSA and 0.125/0.25 µg/mL for MRSA (Fig. 3G).

Vancomycin-intermediate *S. aureus* (VISA) strains exhibit a characteristically thickened cell wall with an increased proportion of non-amidated muropeptides and altered peptidoglycan cross-linking, conferring reduced susceptibility to lysostaphin (25–28). None of the strains in our clinical isolate collection were vancomycin non-susceptible (as defined by MIC ≥ 4 µg/mL), consistent with the low prevalence of vancomycin-intermediate and vancomycin-resistant *S. aureus* in China (29–32). To assess the activity of LYSG101 against strains with reduced vancomycin susceptibility, MICs and MBCs were determined for heterogeneous VISA (hVISA) strain CCUG 45314 and VISA strain ATCC 700699 (MU50) in CAMHB and human serum (Table S1). MICs for CCUG 45314 and ATCC 700699 were 2 and 4 µg/mL in CAMHB, and 0.03125 and 0.125 µg/mL in human serum, respectively. MBCs for CCUG 45314 and ATCC 700699 were 2 and 8 µg/mL in CAMHB, and 0.03125 and 0.25 µg/mL in human serum, respectively. Despite the reduced vancomycin susceptibility of ATCC 700699 (vancomycin MIC = 8 µg/mL), LYSG101 MICs remained within the range observed for vancomycin-susceptible clinical isolates in both CAMHB and human serum (Fig. 3B and F).

**Fig 4:**
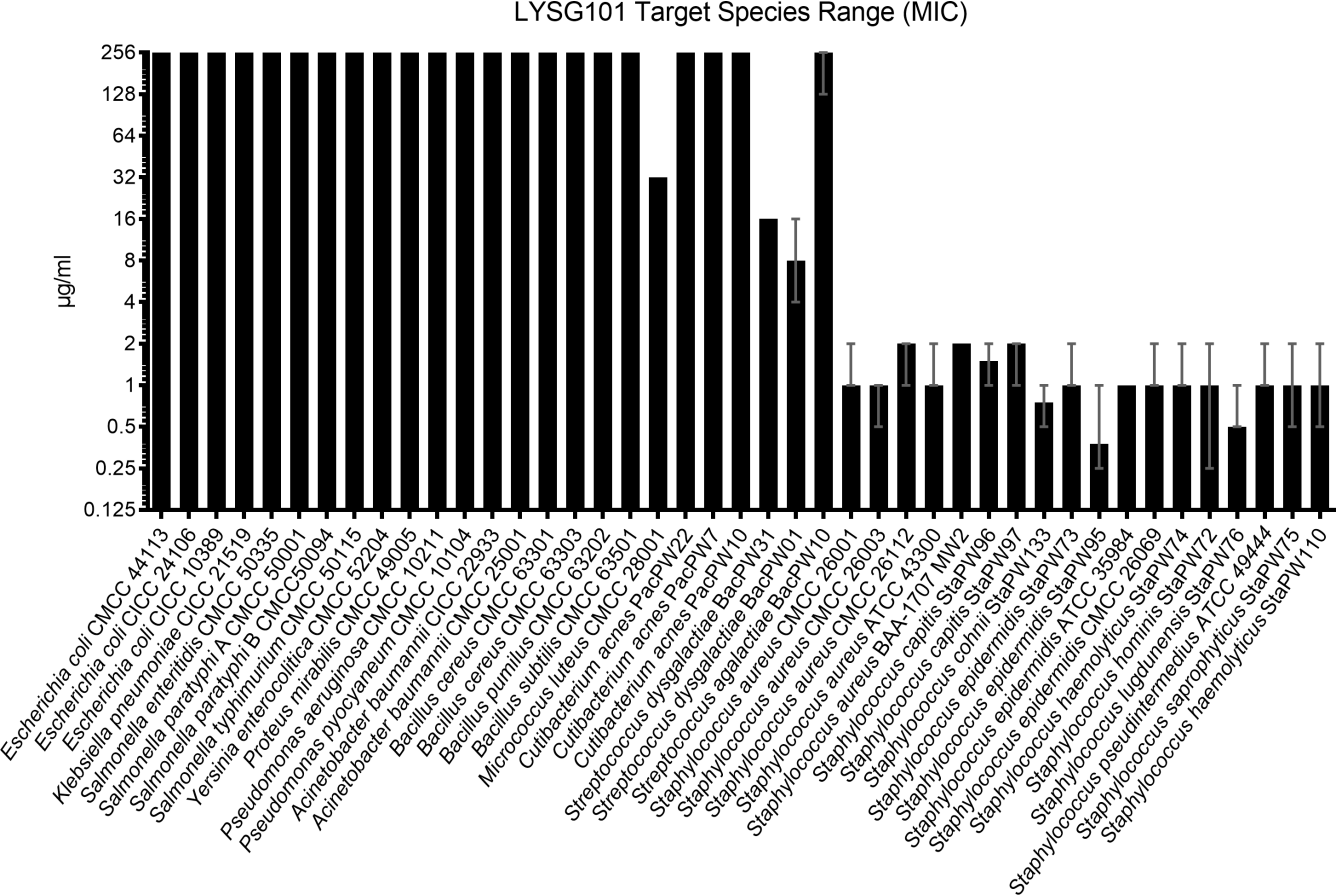
LYSG101 is highly active against *Staphylococcus aureus* and CoNS with minimal activity against other species. LYSG101 MICs were evaluated in CAMHB against a range of bacterial strains as denoted on the *x*-axis using standard CLSI methods. Median MICs were plotted on a log_2_ scale; error bars represent the range (minimum to maximum).

The spectrum of activity of LYSG101 was further evaluated against additional bacterial species using the MIC assay in CAMHB. LYSG101 was inactive against all gram-negative organisms tested, including various *Enterobacteriaceae*, *Pseudomonas aeruginosa*, and *Acinetobacter baumannii* (Fig. 4). Similarly, the enzyme was inactive against *Bacillus subtilis*, *Bacillus cereus, Streptococcus agalactiae*, and *Cutibacterium acnes*, and only weakly active against *Micrococcus luteus* and *Streptococcus dysgalactiae*. For comparison, potent activity was observed against representative isolates of *S. aureus* and CoNS, including *S. capitis*, *S. cohnii*, *S. epidermidis*, *S. haemolyticus*, *S. hominis*, *S. lugdunensis*, *S. pseudintermedius*, and *S. saprophyticus*.

### LYSG101 is bactericidal at sub-MIC concentrations

**Fig 5:**
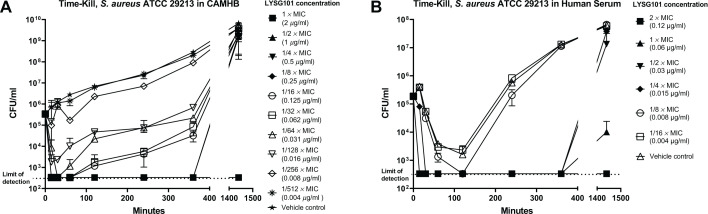
LYSG101 demonstrates potent sub-MIC activity in time-kill assays. Log-phase *Staphylococcus aureus* ATCC 29213 cells were incubated with serially diluted LYSG101 in CAMHB (**A**) or in human serum (**B**) at 37°C with shaking at 200 rpm. At the stated time intervals, samples were removed, LYSG101 was neutralized with activated charcoal/EDTA mixture, and viable CFU were quantified using serial dilution and plating. Error bars represent standard deviation; LYSG101 doses were rounded to three decimal places.

We evaluated sub-MIC killing of *S. aureus* strain ATCC 29213 by LYSG101 using a time-kill assay. In CAMHB, a reduction of >3-log_10_ CFU/mL was observed within 15 min following the addition of LYSG101 (the first time point tested) and was seen over a concentration range of 0.031–2 µg/mL (1/64× MIC – 1× MIC), indicating strong bactericidal activity at sub-MIC concentrations. At the MIC, bacterial CFUs remained undetectable throughout the experiment; however, regrowth was observed at later time points at sub-MIC concentrations (Fig. 5A). CFU/mL reduction of >1-log_10_ was seen at 0.016 µg/mL LYSG101 (1/128× MIC), and minimal to no effect was seen at 0.004 µg/mL (1/512× MIC).

Potent killing at sub-MIC concentrations was also seen in human serum, where the MIC value was substantially lower than that seen in CAMHB (0.06 µg/mL in serum compared to 2 µg/mL in CAMHB for ATCC 29213). A reduction of >3-log_10_ CFU/mL was seen at LYSG101 concentrations of 0.015–0.06 µg/mL (1/4× MIC – 1× MIC), >1-log_10_ CFU/mL killing effect was seen at 0.008 µg/mL (1/8× MIC), and no appreciable effect was seen at 0.004 µg/mL (1/16× MIC) (Fig. 5B).

### Low propensity for resistance development

**Fig 6:**
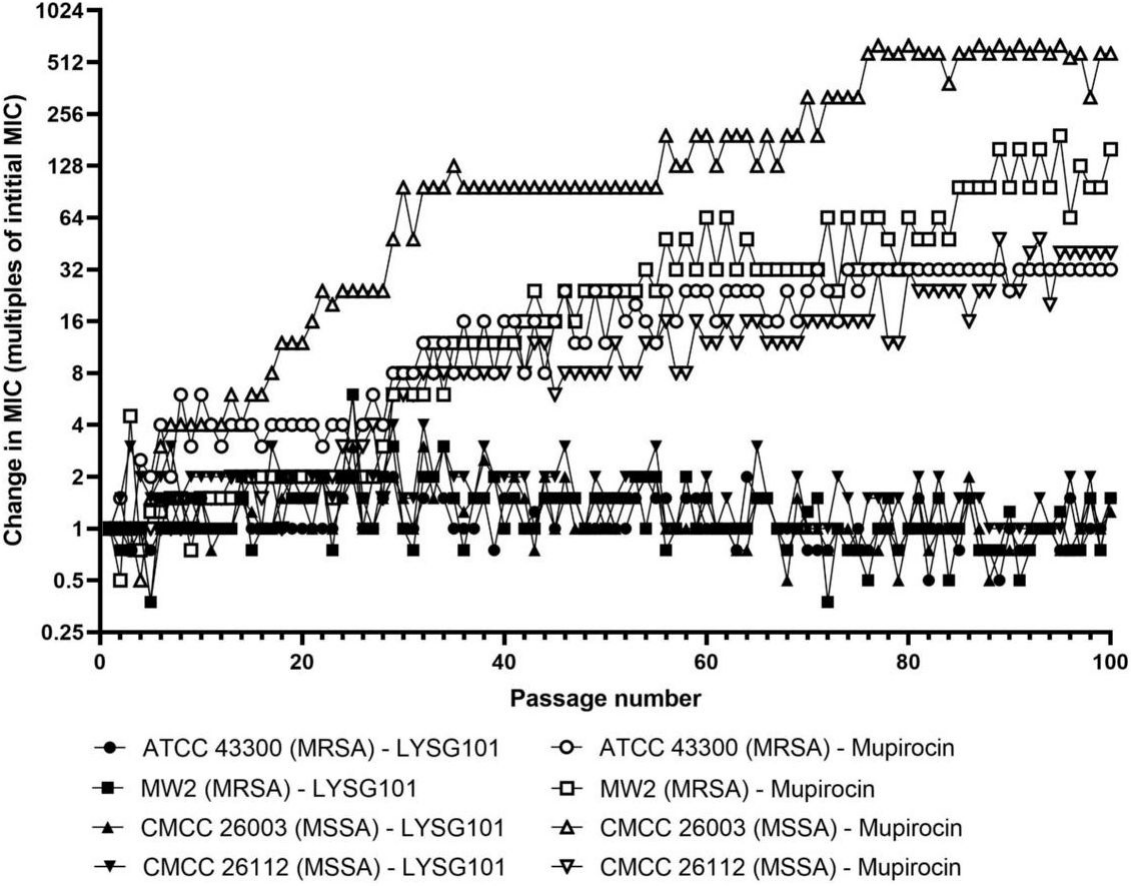
No resistance emerges following prolonged passage in the presence of LYSG101. Two MSSA strains (CMCC 26003 and CMCC 26112) and two MRSA strains (ATCC 43300 and MW2) were each passaged 100 times in duplicate in CAMHB supplemented with twofold serially diluted LYSG101 (black markers) or mupirocin as control (white markers) in 96-well plates. MIC values were expressed as fold change relative to the initial MIC; mean values were calculated from duplicate samples.

To assess the propensity of LYSG101 to select for resistance, we performed a serial passage experiment using two MSSA strains (CMCC 26003 and CMCC 26112) and two MRSA strains (ATCC 43300 and MW2). Each strain was passaged in duplicate in CAMHB with twofold serial dilutions of LYSG101 or mupirocin (positive control), with each subsequent inoculation initiated from the highest sub-inhibitory concentration wells showing bacterial growth. Resistance to mupirocin emerged rapidly across all strains, progressively increasing to 32- to 512-fold higher than the initial concentration by the final passage. In contrast, resistance to LYSG101 did not emerge in any of the strains even after 100 passages, with MIC values remaining unchanged from the initial measurement (Fig. 6).

### LYSG101 rapidly eliminates *S. aureus* and CoNS biofilm

**Fig 7:**
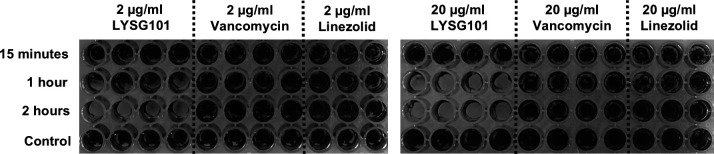
LYSG101 effectively dismantles *Staphylococcus aureus* biofilm. Biofilms of MRSA strain StaPW15 were grown in quadruplicate for 24 h at 37°C in TSB 0.2% glucose in a polystyrene 96-well plate, washed with PBS, and supplemented with TSB 0.2% glucose containing 2 or 20 µg/mL of LYSG101, vancomycin, or linezolid, for 15 min, 1 h, or 2 h. Plates were washed with PBS and treated with crystal violet to visualize biofilms. Also see Fig. S3.

Bacterial biofilms play a pivotal role in chronic staphylococcal infections, often accounting for antibiotic failures (8). We screened >250 *Staphylococcus* strains from our collection to identify those forming the most robust biofilms for use in anti-biofilm studies. A 24 h biofilm of MRSA strain StaPW15, which forms a particularly dense biofilm, was treated with 2 or 20 µg/mL LYSG101, vancomycin, or linezolid, in Tryptic Soy Broth (TSB) supplemented with 0.2% glucose, for 15 min, 1 h, or 2 h. The biofilms were then washed and visualized with crystal violet (Fig. 7). Biofilms were disrupted as early as 1 h after the addition of 20 µg/mL LYSG101. At a concentration of 2 µg/mL, initial effects were observed after 1 h and biofilm disruption was observed 2 h following the addition of LYSG101. In contrast, no biofilm disruption was seen in the wells treated with vancomycin or linezolid. Additional *S. aureus* and CoNS strains forming dense biofilms were similarly evaluated, and rapid biofilm disruption by LYSG101 was observed in all cases (Fig. S3).

### LYSG101 shows no *in vitro* toxicity

To test *in vitro* toxicity, we first evaluated lysis of rabbit red blood cells following exposure to LYSG101. No hemolysis was observed following exposure of the cells to up to 512 μg/mL LYSG101 (the highest concentration tested), while complete lysis was observed with 0.1% Triton X-100, used as a positive control (Fig. S4A). Furthermore, exposure of HaCaT immortalized human epidermal keratinocyte cell line to up to 500 μg/mL LYSG101 (the highest concentration tested) had no discernible effect on cell viability, evaluated using the 3-(4,5-dimethylthiazol-2-yl)−2,5-diphenyltetrazolium bromide (MTT) assay (Fig. S4B).

### *In vivo* efficacy of LYSG101 against *S. aureus* infection

**Fig 8:**
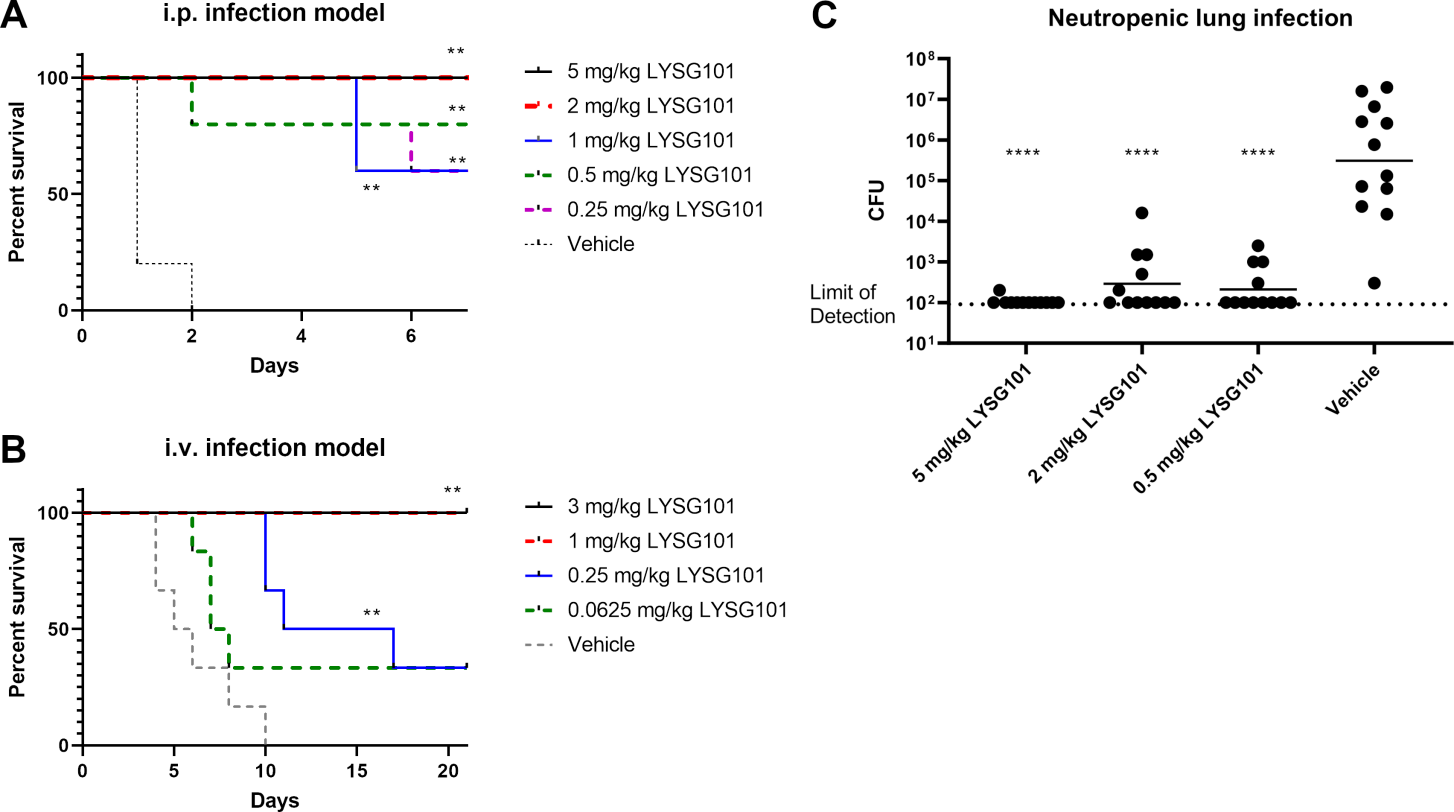
LYSG101 provides effective protection against *Staphylococcus aureus* in intraperitoneal, intravenous, and lung models of infection. (**A**) Six- to 8-week-old female BALB/c mice were infected i.p. with 2 × 10^7^ CFU of log-phase *S. aureus* strain Newman in saline containing 5% mucin. At 2 h post-infection, mice were treated i.p. with the specified dose of LYSG101 or vehicle control. Mice were observed for 1 week for signs of morbidity or death. (**B**) Six- to 8-week-old female BALB/c mice were injected i.v. with 4.4 × 10^7^ CFU of log-phase *S. aureus* strain Newman in saline. At 2 h post-infection, mice were injected i.v. with the specified dose of LYSG101 or vehicle control. Mice were observed for 3 weeks for signs of morbidity or death. Survival graphs were analyzed using the Gehan-Breslow-Wilcoxon test; **P < 0.01 versus vehicle. (**C**) In a neutropenic lung infection model, 6- to 8-week-old female BALB/c mice were made neutropenic by cyclophosphamide administration and infected intranasally with 4.5 × 10^5^ CFU of log-phase *S. aureus* strain Newman in saline containing 3% mucin. At 2 h post-infection, mice were treated intranasally with the specified dose of LYSG101 or vehicle control. At 24 h post-treatment, mice were sacrificed, lungs were homogenized, and CFU were quantified by serial dilution and plating. Data were pooled from two identical experiments performed on different days. Statistical significance was calculated using the Mann-Whitney test; ****P < 0.0001 versus vehicle.

To evaluate the *in vivo* efficacy of LYSG101, three murine infection models were employed: two lethal challenge models and a neutropenic lung infection model. In the first model, mice were injected intraperitoneally (i.p.) with a lethal dose of *S. aureus* in 5% mucin and 2 h later treated i.p. with various doses of LYSG101 or vehicle control. Mice treated with vehicle control quickly succumbed to infection, with none surviving by day 2. LYSG101 demonstrated significant protection across all dosage groups tested (*P* < 0.01), with 5 and 2 mg/kg of LYSG101 providing complete protection, and 1, 0.5, and 0.25 mg/kg resulting in 60–80% protection (Fig. 8A).

In the second model, mice were infected intravenously (i.v.) through the tail vein with a lethal dose of *S. aureus* in saline. At 2 h post-infection, mice were treated i.v. with various doses of LYSG101 or vehicle control. All vehicle control-treated mice succumbed to infection by day 10, whereas mice treated with 3 or 1 mg/kg LYSG101 were completely protected, and mice treated with 0.25 mg/kg showed significant (*P* < 0.01) delay in time to death and 40% survival. While treatment with 0.0625 mg/kg of LYSG101 did not result in a statistically significant effect, it demonstrated a delay in time to death and 40% survival (Fig. 8B).

The efficacy of LYSG101 was further assessed in a neutropenic lung infection model. Following induction of neutropenia with cyclophosphamide, mice were infected intranasally with log-phase *S. aureus* strain Newman in 3% mucin. At 2 h post-infection, mice were treated intranasally with 5, 2, or 0.5 mg/kg LYSG101 or vehicle control and sacrificed 24 h post-treatment. Lung CFU were quantified using homogenization, serial dilution, and plating. In all tested doses, a single administration of LYSG101 resulted in a >3-log_10_ reduction in CFU (*P* < 0.0001 compared to vehicle control), in many cases to below the limit of detection (Fig. 8C).

## DISCUSSION

Lysins represent a promising new class of antimicrobials—direct lytic agents. Unlike traditional antibiotics, lysins are protein therapeutics that actively hydrolyze the bacterial cell wall, causing rapid lysis and cell death. Due to this unique mechanism of action, lysins are targeted to specific pathogens, can effectively degrade biofilms, and are effective against bacteria resistant to traditional antibiotics. Importantly, lysins have a low propensity for resistance (33–37).

Here, we demonstrate that the engineered chimeric lysin LYSG101 has more rapid lysis kinetics than the clinical-stage lysin exebacase. While exebacase is a native streptococcal lysin (17), LYSG101 is a chimeric lysin that shares the same binding domain with exebacase, but has a catalytic domain of staphylococcal origin (15), which likely contributes to its superior lytic activity against staphylococci. Structural modeling further suggests that domain orientation plays an important role in catalytic activity. While the catalytic and binding domains of LYSG101 interact in a manner roughly similar to that seen in native exebacase, in ClyF (a closely related lysin with a shorter linker), the two domains do not interact, and catalytic activity is suboptimal.

LYSG101 exhibited potent antimicrobial activity against a diverse panel of more than 300 clinical isolates of *S. aureus* (MSSA and MRSA) and CoNS. MIC_50/90_ values were 1/2 µg/mL in CAMHB, 0.0625/0.125 µg/mL in CAMHB + 25% horse serum (equivalent to CLSI-approved AST medium for exebacase [24], but without DTT), and 0.03125/0.03125 µg/mL in human serum. The markedly enhanced LYSG101 activity in serum is consistent with results observed for exebacase, where serum and the serum components lysozyme and albumin were shown to potentiate lysin activity (22). We observed similar synergistic effects between LYSG101 and serum, human lysozyme, and human serum albumin. In the exebacase study, DTT was included for better preservation of enzymatic activity in frozen panels (23). As was seen for exebacase, inclusion of DTT resulted in a slight reduction in MICs. In this study, only fresh panels that did not include DTT were used, as DTT is not needed for preservation of activity under these conditions. The low MIC values observed in serum suggest that LYSG101 may be an effective candidate for the treatment of systemic staphylococcal infections. Importantly, LYSG101 showed approximately one order of magnitude lower MIC_50_ and MIC_90_ values than those of exebacase (38, 39).

Bactericidal activity was confirmed by MBC assays, where MBC_50/90_ values in CAMHB were 2/4 µg/mL, only a single dilution above the MIC_50/90_. A >3-log_10_ CFU/mL reduction as early as 15 min after the addition of LYSG101 (the earliest time point evaluated) in time-kill assays further supported bactericidal activity. LYSG101 was highly effective against all staphylococcal strains tested yet showed little to no activity against other bacterial species. This selective action could be important in preserving the commensal microbiota during treatment. While MICs against the VISA strain ATCC 700699 in CAMHB and serum were at the upper end of the range observed for clinical staphylococcal isolates, they remained within the range observed for vancomycin-susceptible *S. aureus* (VSSA) clinical isolates.

LYSG101 showed potent sub-MIC bactericidal activity in time-kill assays in both CAMHB and human serum. In CAMHB, >3-log_10_ CFU/mL killing was seen between MIC (2 µg/mL) and 1/64× MIC (0.031 µg/mL), with killing gradually diminishing until reaching the baseline at approximately 1/512× MIC (0.004 µg/mL). In serum, >3-log_10_ CFU/mL killing was seen between MIC (0.06 µg/mL) and 1/4× MIC (0.015 µg/mL), and activity reached the baseline at 1/16× MIC (0.004 µg/mL). These data indicate that LYSG101 retains bactericidal activity at concentrations well below the MIC. Although serum strongly synergizes with LYSG101, resulting in a meaningful reduction in MIC, the killing activity returned to baseline at approximately the same LYSG101 concentration in both CAMHB and serum.

It has been hypothesized that lysins have a low propensity for resistance selection due to the co-evolution of phages with their hosts to target conserved cell wall components that are not readily subject to mutational alteration (33, 40). In most studies, 30 serial passages were considered sufficient to evaluate the propensity for the emergence of resistance (41–43). Here, we performed 100 serial passages and observed no change in LYSG101 MIC in any of the strains tested. In contrast, resistance to mupirocin was readily achieved by serial passaging, reaching up to 500-fold above the original MIC. The remarkably low tendency for resistance development observed for LYSG101 may support its use as a first-line antimicrobial treatment. This characteristic is also crucial for managing chronic conditions such as osteomyelitis, where sustained efficacy is vital to prevent treatment failure and promote long-term recovery (44).

Biofilm formation is a key aspect of complex infections, ranging from catheter-related infections to endocarditis and prosthetic joint infections (8). Lysins operate through a mechanism distinct from antibiotics, effectively lysing bacteria within the biofilm matrix and disassembling the biofilm structure (36, 45–47), and have shown promise in treating staphylococcal PJI (13). In this study, we evaluated the activity of LYSG101 against a collection of *S. aureus* and CoNS strains producing dense biofilms, including antibiotic-resistant variants. LYSG101 effectively dismantled biofilms formed by all evaluated strains. LYSG101 was also highly effective *in vivo*, showing complete protection in i.p. and i.v. murine infection models at 2 and 1 mg/kg, respectively, and significant protection and delay in time to death at meaningfully lower concentrations. Additionally, in a neutropenic lung infection model, a >3-log_10_ CFU reduction was observed in the lungs of mice following a single intranasal administration of LYSG101 at doses as low as 0.5 mg/kg. These results appear to compare favorably with other lysins in clinical development for staphylococcal infections (48, 49).

One limitation of this study is that the clinical strain collection used to evaluate LYSG101 activity was geographically restricted, limited primarily to China, and was collected before 2019. Additionally, this study evaluated only a single hVISA and a single VISA strain, neither of which was derived from the clinical panel evaluated. We plan to expand this study to include additional regions and more contemporary isolates and specifically address antibiotic resistance patterns more prevalent in regions outside China. Nevertheless, we believe that the conserved nature of the cell wall target of LYSG101 and the complete absence of resistant isolates in the data set suggest that the general trends observed are likely reproducible. Another limitation arises from the fact that the method used for the commercial production of exebacase has not been published; therefore, we used the method described by Gilmer et al. (17). Although the final product is theoretically similar, the results comparing LYSG101 and exebacase may not fully reflect the characteristics of commercial-grade exebacase. Future studies will focus on further preclinical and clinical development of LYSG101, examining its efficacy in more complex animal models, as well as conducting a comprehensive safety and toxicology evaluation.

In conclusion, we have demonstrated that LYSG101 is a promising antibacterial agent for treating antibiotic-resistant staphylococcal infections, exhibiting markedly higher potency compared to exebacase. Its favorable activity profile, low MIC and MBC values, rapid bactericidal action, absence of resistance, effectiveness against biofilms, and *in vivo* efficacy position it as a suitable candidate for further development to address the growing challenge of antibiotic-resistant staphylococcal infections.

## MATERIALS AND METHODS

### OD reduction

An isolated colony of *S. aureus* CMCC 26003 was transferred to 5 mL TSB in a 50 mL tube and grown overnight at 37°C with shaking at 200 rpm. The overnight culture was diluted 1:100 into 500 mL TSB in a 2 L shake flask and grown at 37°C with shaking at 200 rpm until an OD_600_ of 0.5 was reached. The cells were harvested, washed with phosphate-buffered saline (PBS), resuspended in PBS supplemented with 25% glycerol to a final OD_600_ of 48.0, and frozen in aliquots at −80°C. Prior to each experiment, an aliquot was thawed, washed, and suspended in cation-adjusted Tris-buffered saline (CATBS, 20 mM Tris, 0.9% NaCl, 0.2 g/L CaCl_2_, and 0.2 g/L MgCl_2_, pH 7.4) to an OD_600_ of 1.0. Lysins were serially diluted in CATBS in a 96-well plate, 100 µL/well. To each well, 100 µL of the washed bacteria was added with a multichannel pipette, and the plate was immediately placed in a Multiskan GO plate reader (Thermo Scientific, Waltham, MA, USA) at 37°C, set to read the OD_600_ values of all wells every minute for 15 min with 5 s shaking between reads. Assays were conducted in triplicate, and all plates included a LYSG101 standard and vehicle control wells.

### MIC and MBC assays

MIC assays were conducted according to CLSI document M07-A10, January 2015 (50). Briefly, 96-well plates were prepared with twofold serial dilution of LYSG101 in 100 µL/well growth matrix (CAMHB, CAMHB + 25% horse serum, or human serum). Specific wells were dedicated to growth control (no LYSG101) and sterility control (200 µL/well assay matrix only). Bacteria were streaked on a tryptic soy agar (TSA) plate and incubated at 35 ± 2°C for 18–24 h to obtain isolated colonies. Colonies were scraped from the plate with a sterile loop, suspended in 5 mL of saline, and the suspension was adjusted to a 0.5 McFarland standard. This bacterial inoculum was further diluted 1:50 in the assay matrix, and 100 µL of the diluted bacterial suspension was added to each well except the sterility control. Plates were incubated for 16–20 h at 35 ± 2°C and MICs were interpreted according to the CLSI manual. All experiments were conducted with quadruplicate samples. MBC assays were performed according to CLSI document M26-A (51). MBC values were defined as the lowest concentration of lysin that provided a 3-log_10_ reduction in CFU compared to the initial bacterial inoculum, determined by quantitative plating following 16–20 h incubation at 35 ± 2°C.

### Time-kill assays

The bactericidal activity of LYSG101 was evaluated according to the method described in CLSI document M26-A (51), with some modifications. *S. aureus* strain ATCC 29213 was inoculated from a single colony and cultured overnight in CAMHB at 37°C with shaking at 200 rpm. The culture was diluted 1:100 in CAMHB and incubated at 37°C until an OD_600_ of 0.5 was reached. Subsequently, the culture was diluted in CAMHB to an OD_600_ of 0.1 and further diluted 1:50 in the assay matrix. Bacterial cells were mixed 1:1 with twofold serially diluted LYSG101 in the assay matrix, resulting in approximately 5 × 10^5^ CFU in a final volume of 1 mL within a 5 mL tube. The tubes were incubated at 37°C with shaking at 200 rpm. Samples were taken at various time points: just before the addition of LYSG101, at 15 and 30 min, and 1, 2, 4, 6, and 24 h post-addition, and LYSG101 activity was immediately neutralized by diluting 1:10 in PBS containing 2% activated charcoal and 10 mM EDTA, which had been confirmed not to affect bacterial viability. CFU quantification was performed through serial 10-fold dilutions and plating. Experiments were performed in duplicate; bactericidal activity was defined as a decrease of ≥3-log_10_ CFU/mL compared to the initial inoculum.

### Time-lapse microscopy

*S. aureus* ATCC 29213 was inoculated from a single colony and cultured overnight in TSB at 37°C with shaking at 200 rpm. The culture was diluted 1:100 in TSB, incubated at 37°C until an OD_600_ of 0.5 was reached, and the cells were then washed and resuspended in a similar volume of PBS. The cells were attached to a poly-l-lysine-coated 35 mm glass-bottomed plate for 10 min, washed with PBS, and covered with 4 mL PBS. The plate was placed on the stage of a Nikon Eclipse Ti microscope with a DS-Ri2 camera (Nikon, Tokyo, Japan) and an Olympus UPlanSApo 100×/1.40 oil-immersion lens (Olympus, Tokyo, Japan) coupled with a 1.5× optovar. Differential interference contrast (DIC) live image capture was initiated, and LYSG101 was added to a final concentration of 100 µg/mL.

### Serial passage resistance assay

Emergence of resistance was evaluated with a procedure based on Berti et al. (41). Bacterial strains were subcultured on TSA plates, and two isolated colonies per strain were grown in TSB at 35 ± 2°C overnight and used as the initial inoculum. For each of 100 serial daily passages, a 96-well plate was prepared with twofold serially diluted LYSG101 or mupirocin in CAMHB and included growth and sterility controls. The well with the highest concentration of the test agent permitting bacterial growth in each passage was diluted 1:500 in CAMHB and used to inoculate the next passage as well as to prepare a glycerol stock for storage at −80°C. Where an increase in MIC was observed, the test agent concentration was increased accordingly in subsequent passages.

### Biofilm eradication assays

Biofilm assays were carried out with slight modifications to the protocol of Schuch et al. (46). Bacterial isolates were subcultured on TSA plates and grown overnight at 37°C. Colonies were resuspended in saline to an OD_600_ of 0.1, diluted 1:1,000 in TSB + 0.2% glucose, and 100 µL of the resulting suspension was dispensed in triplicate or quadruplicate into each well of a 96-well microtiter plate. The plates were incubated at 37°C for 24 h to allow biofilm formation and washed three times with PBS. Then, 100 µL of TSB + 0.2% glucose containing either 2 or 20 µg/mL of LYSG101 (or where applicable, vancomycin or linezolid comparators) was added to each well and incubated at 37°C for 15 min, 1 h, or 2 h, and then washed three times with PBS. The biofilms were stained by adding 100 µL of 0.05% crystal violet solution for 15 min, washed three times with PBS, and photographed.

### Animal models

Mice were obtained from Myhalic Biotechnology Co., Ltd., and housed at 25–27°C, 40–60% humidity, under a 12 h light-dark cycle, with standard rodent chow and water provided *ad libitum*.

Bacteria were prepared for animal experiments as follows: an isolated colony of *S. aureus* strain Newman was inoculated into 5 mL brain heart infusion (BHI) in a 50 mL tube and incubated overnight at 37°C with shaking at 200 rpm. The overnight culture was diluted 1:100 into two 50 mL tubes each containing 5 mL BHI and grown at 37°C with shaking at 200 rpm until an OD_600_ of 0.5 was reached. Cells were then washed once with saline and resuspended in saline to a final OD_600_ of 1.0.

The intraperitoneal infection model was performed according to Raz et al. with slight modifications (52). Washed log-phase bacteria were diluted to 3–4 × 10^7^ CFU/mL in saline containing 5% mucin, which promotes the effective establishment of peritoneal infection (53), and 0.5 mL of this suspension was injected i.p. into 6- to 8-week-old female BALB/c mice (5 mice per group). At 2 h post-infection, mice were treated i.p. with 5, 2, 1, 0.5, or 0.25 mg/kg LYSG101 or vehicle control with a total volume of 500 µL (single dose treatment); it was previously reported that bacteria spread systemically as early as 1 h post-infection in this model (54). Mice were observed daily for 7 days for signs of morbidity (persistent hunched posture, labored breathing, or complete inability to access food and water independently) or mortality.

For intravenous infection, 100 µL of washed log-phase *S. aureus* Newman in saline (prepared as described above) was injected i.v. (through the tail vein) into 6- to 8-week-old female BALB/c mice (6 mice per group), resulting in an infectious dose of 4.4 × 10^7^ CFU per mouse. At 2 h post-infection, mice were injected i.v. with 3, 1, 0.25, or 0.0625 mg/kg LYSG101 or vehicle control with a total volume of 200 µL (single dose treatment). Mice were observed daily for 3 weeks for signs of morbidity or mortality.

The neutropenic lung infection model was performed with slight modifications from Crandon et al. (55), with six 6- to 8-week-old female BALB/c mice per group per experiment (results were aggregated from two identical experiments). Neutropenia was induced by intraperitoneal administration of 150 mg/kg cyclophosphamide at 4 days and 100 mg/kg cyclophosphamide at 1 day prior to infection. Log-phase *S. aureus* Newman bacteria were prepared as described above and diluted to 1.5 × 10^7^ CFU/mL in saline containing 3% mucin. Infection was carried out under isoflurane anesthesia, by administering 30 µL of the bacterial suspension intranasally (4.5 × 10^5^  CFU/mouse). At 2 h post-infection, the mice were again anesthetized and treated intranasally once with 5, 2, or 0.5 mg/kg LYSG101 in a total volume of 50 µL PBS, or 50 µL vehicle control. At 24 h post-treatment, the mice were euthanized with carbon dioxide, and the lungs were placed in a 2-mL tube containing one 5-mm zirconia bead (Sinoshine, Zibo, China), ten 2-mm beads, and 1.3 mL PBS containing 2% activated charcoal and 10 mM EDTA. The lungs were then homogenized with a KZ-III-96 bead-beater tissue homogenizer (Servicebio, Wuhan, China) at 70 Hz for 5 cycles of 45 s each with a 15 s break between cycles. The lung homogenate was serially diluted 10-fold in saline and streaked onto MSA plates to quantify CFU.

### Three-dimensional structural prediction

Three-dimensional structural prediction was performed on an Ubuntu 22.04 server equipped with an RTX3090 GPU, with the ColabFold implementation of AlphaFold2 with default parameters (56). The highest-scoring model for each protein was selected, and images were generated with PyMOL (Molecular Graphics System, Version 3.0, Schrödinger, LLC).

### Statistical analysis

MIC data analysis was performed with Python 3.13.2 and the pandas library (v2.2.3), and visualizations were generated with matplotlib (v3.10.0). Other data were analyzed with GraphPad Prism 8.3.0 for Windows (GraphPad Software, San Diego, CA, USA). Survival data were plotted as Kaplan-Meier curves and each treatment group was compared pairwise with the vehicle control using the log-rank (Gehan-Breslow-Wilcoxon) test. Differences in lung CFU counts between treatment and vehicle groups were analyzed using the Mann-Whitney test. For all statistical tests, a *P* value of <0.05 was considered significant.

## SUPPLEMENTAL MATERIAL

Supplemental material aac.01730-25-s0001.pdf Supplemental methods; Fig. S1 to S5; Tables S1 to S3.

Video S1 aac.01730-25-s0002.mp4 Rapid lysis of *S. aureus* by LYSG101 visualized by video microscopy.

Video S2 aac.01730-25-s0003.mp4 Real-time visualization of bacterial clearing by LYSG101 in liquid.

ASM does not own the copyrights to Supplemental Material that may be linked to, or accessed through, an article. The authors have granted ASM a non-exclusive, world-wide license to publish the Supplemental Material files. Please contact the corresponding author directly for reuse.
