# Supplementary material for: LYSG101: a potent chimeric lysin with therapeutic potential for combating Staphylococcus aureus infections
Source: Antimicrob Agents Chemother. 2026 Apr 30;70(6):e01730-25. doi: 10.1128/aac.01730-25 (PMC13231913; doi:10.1128/aac.01730-25)
Supplement: Supplemental material — Supplemental methods; Fig. S1 to S5; Tables S1 to S3. [file aac.01730-25-s0001.pdf]

SUPPLEMENTAL METHODS

Bacterial strains

Staphylococcal strains and their sources are detailed in Table S2, and other bacterial strains are detailed in Table S3. To ensure purity and identity, each isolate was subjected to two consecutive isolation streaks on Mannitol Salt Agar (MSA) for staphylococcal isolates, MacConkey agar for Gram-negative isolates, Reinforced Clostridial Agar (RCA) for *Cutibacterium acnes*, or Tryptic Soy Agar (TSA) for other isolates. Bacteria were evaluated by Gram-staining and Gram-positive cocci were also evaluated by catalase test. Unless obtained from a qualified repository, *Staphylococcus aureus* strains were also subjected to protein A typing using primers PA 1095F (5'-AGACGATCCTTCGGTGAGC-3') and PA 1517R (5'-GCTTTTGCAATGTCATTTACTG-3') according to Shopsin *et al.* (1). Strains without an amplified protein A gene were excluded from analysis. Methicillin susceptibility was determined using the Kirby-Bauer disk diffusion method with cefoxitin disks according to CLSI guidelines in document M100 (2). Vancomycin MIC was evaluated in cation-adjusted Mueller-Hinton Broth (CAMHB) according to CLSI document M07-A10, January 2015 (3); presence of heterogeneous vancomycin intermediate *S. aureus* (hVISA) was not evaluated.

Escherichia coli strains were grown in lysogeny broth (LB) at 37°C with shaking at 200 rpm or on LB agar plates; tryptone and yeast extract were from Oxoid (Basingstoke, Hants, UK). Where appropriate, media were supplemented with ampicillin (100 µg/mL) or kanamycin (50 µg/mL). *Cutibacterium acnes* was cultured in Reinforced Clostridial Medium (RCM; prepared according to a recipe from Oxoid, with components sourced from Oxoid and SCR, Shanghai, China) at

37°C under an atmosphere of 80% nitrogen, 10% carbon dioxide, and 10% hydrogen. Other bacterial strains were grown on TSA at 37°C or in Tryptic Soy Broth (TSB; BD, Becton, Dickinson and Company, Franklin Lakes, NJ, USA), or when specified, in CAMHB (BD, USA) or Brain Heart Infusion (BHI; BD, USA) at 37°C with shaking at 200 rpm.

Reagents

Phosphate-buffered saline (PBS) and cation-adjusted Tris-buffered saline (CATBS) were prepared from analytical grade reagents purchased from SCR (Shanghai, China). Poly-L-lysine was purchased from Solarbio (Cat. No. P8141, Shanghai, China), mupirocin was from Ourchem (Shanghai, China), and Mucin (Cat. No. M2378-100G) was from Sigma-Aldrich. Human serum (Cat. No. H4522-100mL) was from Sigma-Aldrich, and horse serum (Cat. No. S9050) was from Solarbio. Other reagents not otherwise specified were obtained from SCR.

Plasmid construction

Restriction enzymes were from New England Biolabs (Ipswich, MA, USA) and T4 DNA Ligase was from Thermo Fisher Scientific (Waltham, MA, USA). DNA purification was performed with commercial kits from Omega Bio-tek (Norcross, GA, USA). Open reading frames of genes used in this study were produced via gene synthesis (Sangon, Shanghai, China) and inserted into pET28a using restriction enzyme digestion and ligation, such that gene products were produced in native form without purification tags. Plasmid pPW-LYSG101 was designed according to Wei *et al.* (4) and the amino acid sequence and domain organization are presented in Fig. S5A. Plasmid pPW-ClyF was designed according to the sequence provided in the supplemental material of Yang

et al. (5), and the amino acid sequence and domain organization are presented in Fig. S5B. pPW-Exebacase was designed according to Gilmer *et al.* (6), NCBI accession number ZP_03625529.1; the domain organization was previously described by Schuch *et al.* (7). Plasmid pPW-LYSG101-C36S is similar to pPW-LYSG101; however, nucleotide 107 in the open reading frame was changed from G to C, such that the codon TGT encoding cysteine was replaced with TCT encoding serine. Plasmids were transformed into *E. coli* DH5α, and the insert sequence was verified by Sanger sequencing of both strands. Verified plasmids were extracted and transformed into *E. coli* BL21(DE3) for protein expression.

Protein purification

E. coli BL21(DE3) cells containing the respective expression plasmids were diluted from an overnight culture 1:100 into ZYM-5052 medium (8), grown for 3 h at 37°C with shaking at 300 rpm and then overnight at 20°C with shaking at 300 rpm. Cells were harvested by centrifugation and lysed by high-pressure homogenization. For LYSG101, LYSG101-C36S, and ClyF, the soluble protein fraction was loaded on a Capto MMC column (Cytiva, Sweden) and eluted with a pH and salt gradient. The eluted fraction was loaded on a fast-flow phenyl Sepharose column (Cytiva) and eluted with a salt gradient. Endotoxins and remaining impurities were removed by passage through a fast-flow Q-Sepharose column (Cytiva). Exebacase (PlySs2) was purified as described previously (6).

Real-time visualization of bacterial clearing in liquid

An overnight culture of *S. aureus* CMCC 26003 was diluted 1:100 in TSB, incubated at 37°C with shaking at 200 rpm until an OD_{600} of 0.7 was reached. Bacteria were washed and resuspended in CATBS to an OD_{600} of 1.0 and divided into two T-25 Corning flasks (30 mL each). LYSG101 was added to one of the flasks to a final concentration of 50 µg/mL and turbidity reduction was recorded by video. A similar volume of vehicle was added to the control flask before imaging.

Checkerboard assay

Checkerboard assays were performed with slight modifications from Moody *et al.* (9). LYSG101 was initially added to well A1 at 4× the final concentration and to wells B1 to H1 at 2× the final concentration. It was then serially diluted twofold from column 1 to column 9 and excess volume from column 9 was discarded; column 10 contained no LYSG101. In separate experiments, serum, albumin, or lysozyme was prepared at 4× the final concentration in CAMHB and added 1:1 to wells A1 to A10 (resulting in 2× the final concentration of both LYSG101 and the second agent in row A). The contents of row A were then serially diluted twofold from row A to row G, and excess volume from row G was discarded; row H contained LYSG101 only. Bacterial working inoculum of MRSA strain BAA-1707 (MW2) was prepared in CAMHB as described for the MIC method, and added 1:1 to each well (i.e., 100 µL each), resulting in a final volume of 200 µL in each well and the stated 1× concentration for each agent in each of the assay wells (final concentration of each agent is listed in Fig. S1). Column 11 and well H10 contained CAMHB and bacterial inoculum only (no test agents) and were used as growth controls. Column 12 contained CAMHB only and was used as a sterility control. The plates were incubated for 16-20 h at 35 ± 2°C.

Rabbit blood hemolysis assay

Red blood cells were prepared by centrifuging rabbit whole blood in sodium citrate (Hongquan Bio, Guangzhou, China) at 1000 × *g* at 4°C for 5 min. The cell pellet was washed and resuspended in saline at five times the original blood volume. A twofold dilution series of LYSG101 was prepared, ranging from 1024 μg/mL to 0.5 μg/mL in 20 mM sodium phosphate, 150 mM NaCl, pH 7.4. Equal volumes (100 μL + 100 μL) of washed red blood cells and serially diluted LYSG101 were mixed in a 96-well plate and shaken at 300 rpm for 3 min. Dilution buffer (20 mM sodium phosphate, 150 mM NaCl, pH 7.4) served as a negative control and 0.1% Triton X-100 served as a positive control. The plate was incubated stationary at 37°C for 1 h, centrifuged at 800 × *g*, at 4°C for 10 min, and 100 μL of the supernatant was transferred to a new plate for absorbance measurement at 405 nm. The reactions were carried out in triplicate and the data are shown as means ± standard deviations.

MTT cell viability assay

Cell viability assays were performed using the HaCaT immortalized human epidermal keratinocyte cell line, obtained from Fenghui Bio (Hunan, China). Cells were cultured in Dulbecco's modified Eagle medium (DMEM; Fenghui Bio) supplemented with 10% fetal bovine serum (Fenghui Bio), 100 μg/mL streptomycin, and 100 U/mL penicillin at 37°C in a humidified atmosphere containing 5% CO_2. Cells were seeded into 96-well plates at a density of 2×10^4 live cells per well and cultured for 3 days. The culture medium was then removed, and the cells were treated with LYSG101 in saline at concentrations of 0, 50, 100, 250, and 500 μg/mL for 1 h at

37°C in 5% CO_2. After treatment, the LYSG101 solution was removed, and the cells were incubated with 0.5 mg/mL MTT in culture medium for 3 h at 37°C in 5% CO_2. The culture medium was then removed, and 100 μL of dimethyl sulfoxide (DMSO) was added to each well. The plate was shaken at 800 rpm for 1 min to solubilize formazan crystals, and absorbance was measured at 570 nm. Percent viability was calculated as follows: (absorbance of LYSG101-treated cells) / (absorbance of vehicle-treated cells) × 100%. All assays were performed in quadruplicate, and the data are presented as means ± standard deviations.

SUPPLEMENTARY FIGURES

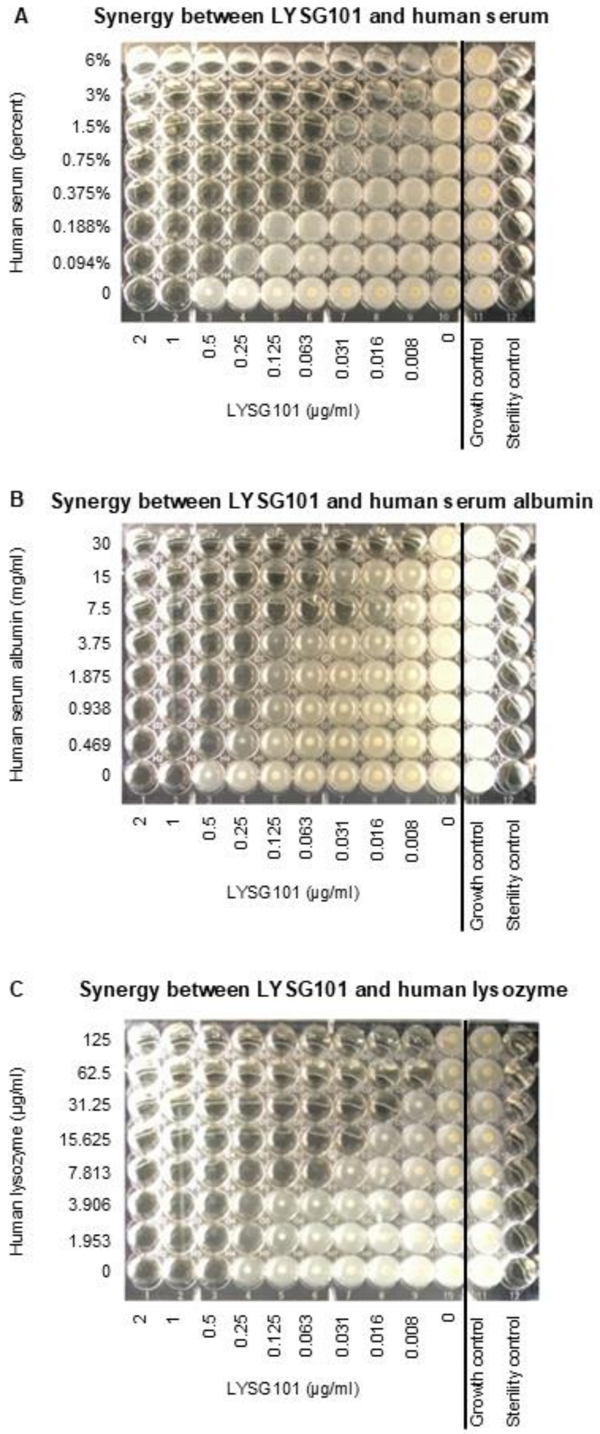

FIG S1 Synergy between LYSG101 and human serum, human serum albumin, and human lysozyme. Checkerboard microwell plates were prepared with LYSG101 diluted 2-fold from column 1 to 9. Human serum (A), human serum albumin (B), or human lysozyme (C), was serially diluted 2-fold from row A to row G. Column 10 contained no LYSG101 and row H did not contain serum/albumin/lysozyme. Column 11 was used as a growth control and column 12 was used as a sterility control. A working inoculum of MRSA strain BAA-1707 (MW2) was added to each well, resulting in a final volume of 200 µL and a final concentration for each test agent as denoted in the figure. Plates were incubated for 16 to 20 h at 35 ± 2°C.

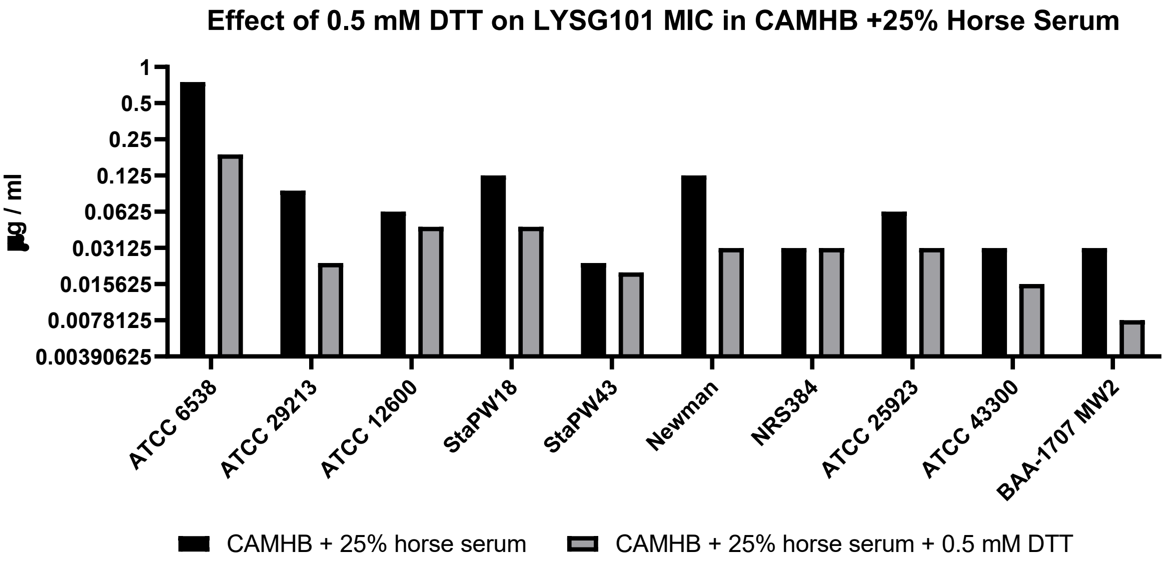

FIG S2 Effect of 0.5 mM DTT on LYSG101 MIC in CAMHB + 25% horse serum. MIC assays were conducted according to CLSI procedures in CAMHB + 25% horse serum with or without 0.5 mM DTT. Strains representing a range of LYSG101 MIC values were selected for evaluation.

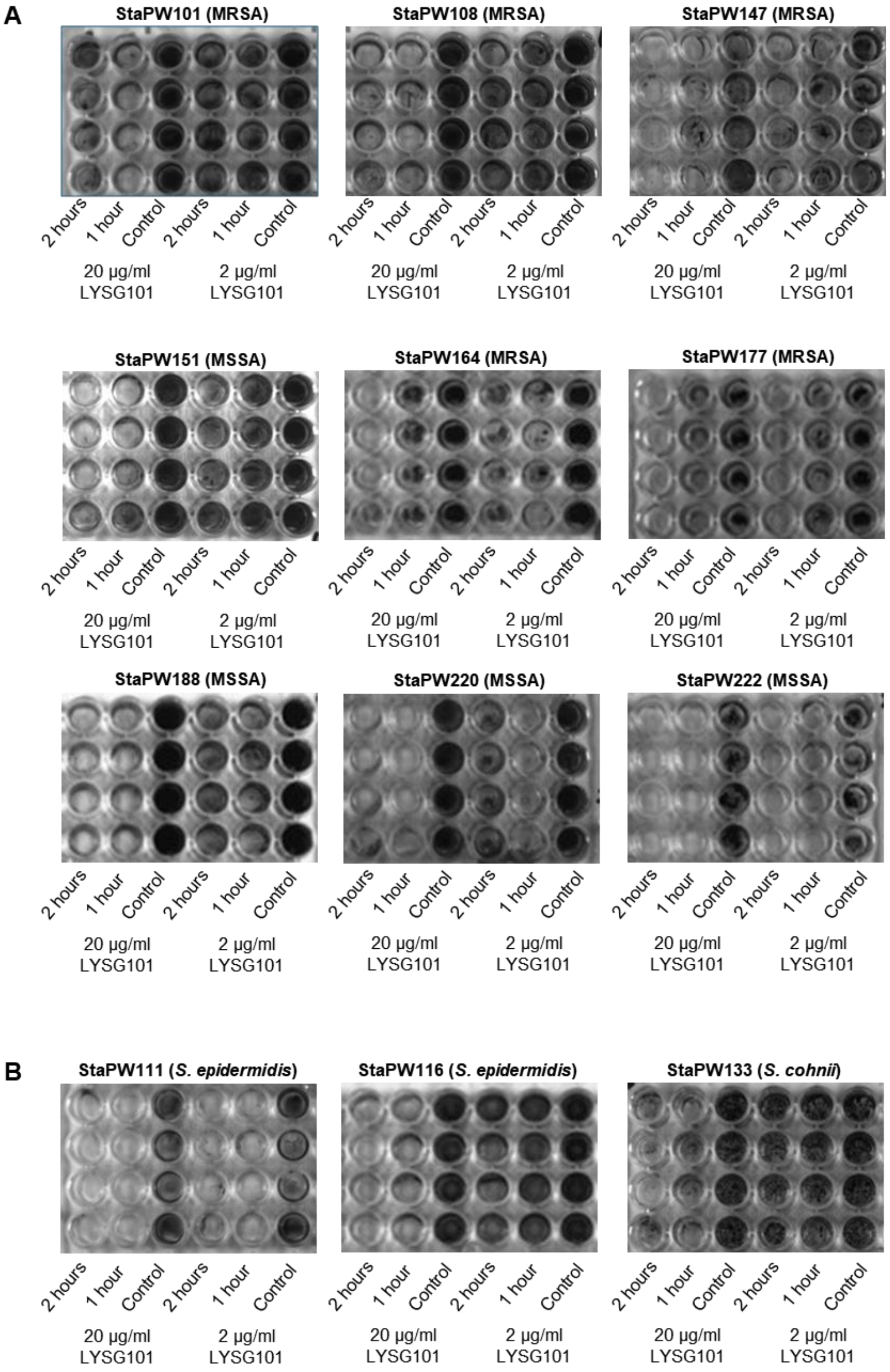
A
StaPW101 (MRSA)
StaPW108 (MRSA)
StaPW147 (MRSA)
StaPW151 (MSSA)
StaPW164 (MRSA)
StaPW177 (MRSA)
StaPW188 (MSSA)
StaPW220 (MSSA)
StaPW222 (MSSA)
B
StaPW111 (S. epidermidis)
StaPW116 (S. epidermidis)
StaPW133 (S. cohnii)
2 hours
1 hour
Control
2 hours
1 hour
Control
20 µg/ml LYSG101
2 µg/ml LYSG101

FIG S3 LYSG101 effectively dismantles *S. aureus* and CoNS biofilms. Biofilms of *S. aureus* MSSA and MRSA strains (A), and CoNS strains (B), were grown in quadruplicate for 24 h at 37°C in TSB 0.2% glucose in a polystyrene 96-well plate, washed with PBS, and supplemented with TSB 0.2% glucose containing 2 µg/mL or 20 µg/mL LYSG101 for 1 or 2 h. Plates were washed with PBS and treated with crystal violet to visualize biofilms.

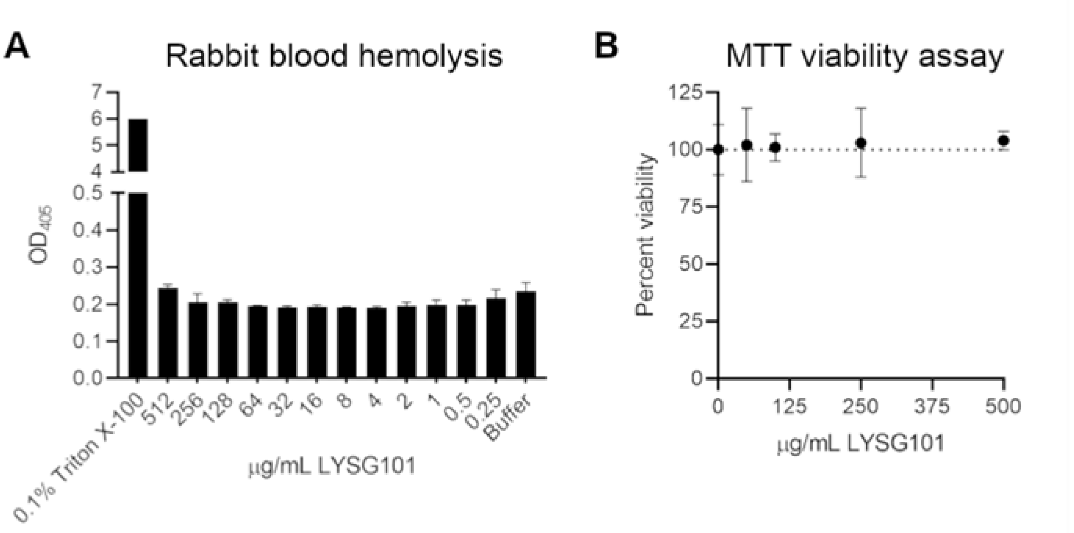

FIG S4 *In vitro* evaluation of LYSG101 toxicity. Washed rabbit red blood cells were mixed with 2-fold serially diluted LYSG101 or 0.1% Triton X-100 (positive control) for 1 h at 37°C. Release of hemoglobin into the supernatant was evaluated by measuring absorbance at 405 nm (A). HaCaT cell viability was evaluated using the MTT assay. A monolayer of HaCaT cells was treated with twofold serially diluted LYSG101 in saline for 1 h at 37°C. The cells were washed and formazan crystals were solubilized with DMSO and evaluated by absorbance at 570 nm. Percent viability is defined as (absorbance of LYSG101-treated cells) / (absorbance of vehicle-treated cells) × 100% (B).

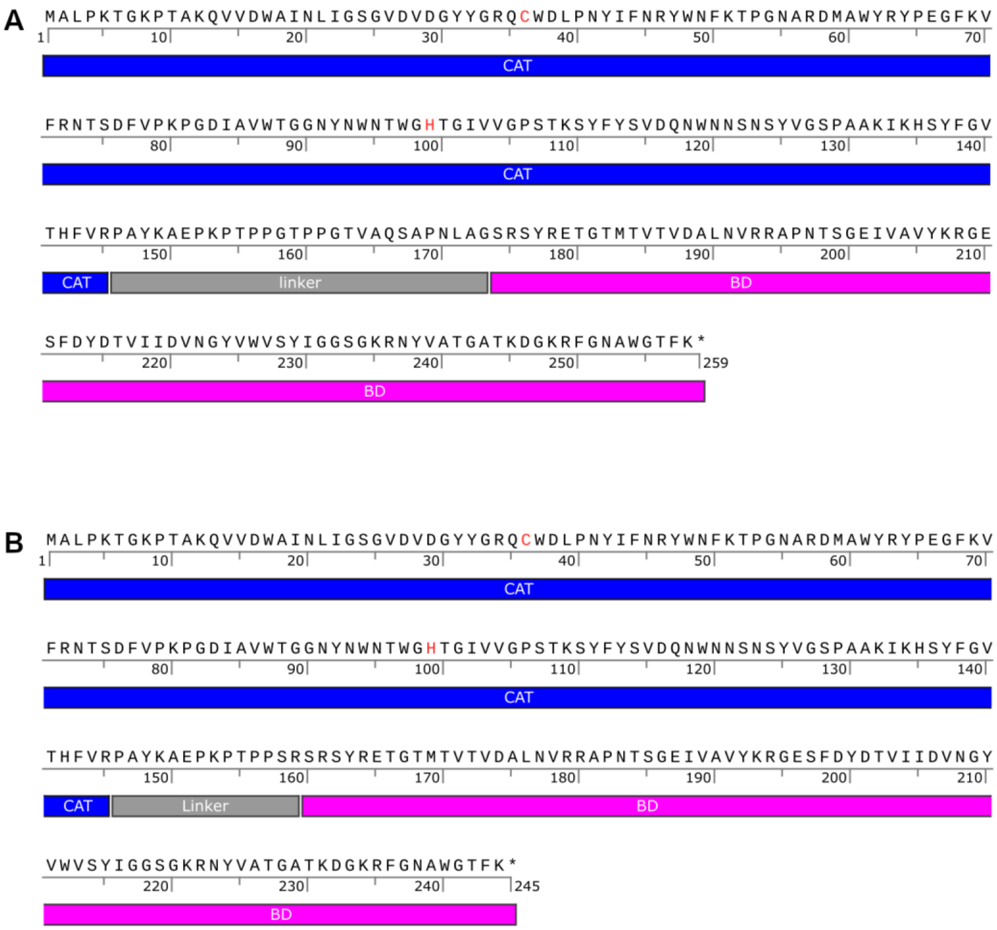

FIG S5 Domain organization of LYSG101 (ClyO) and ClyF. Amino acid sequences of LYSG101 (A) and ClyF (B) are presented along with domain organization. Catalytic domain (CAT) is presented in blue, and binding domain (BD) is presented in magenta. Active-site Cysteine and Histidine residues are colored red.

SUPPLEMENTARY TABLES

TABLE S1

Strain	Designation	Vancomycin MIC	LYSG101			
			CAMHB		Human serum	
			MIC	MBC	MIC	MBC
CCUG 45314	hVISA	2 µg/ml	2 µg/ml	2 µg/ml	0.03125 µg/ml	0.03125 µg/ml
ATCC 700699 (MU50)	VISA	8 µg/ml	4 µg/ml	8 µg/ml	0.125 µg/ml	0.25 µg/ml

TABLE S1 - LYSG101 MICs and MBCs for Vancomycin-Intermediate *S. aureus* (VISA) and heterogeneous VISA (hVISA).

TABLE S2 – *Staphylococcus strains*

Strain	Source	Organism name	Ridom	Rtype
**ATCC 43300**	ATCC	*Staphylococcus aureus*	NP	NP
**ATCC 700699 (MU50)**	Mingzhou	*Staphylococcus aureus*	NP	NP
**BAA-1707 MW2**	ATCC	*Staphylococcus aureus*	NP	NP
**CCUG 45314**	BNCC	*Staphylococcus aureus*	NP	NP
**CMCC 26001**	CMCC	*Staphylococcus aureus*	NP	NP
**CMCC 26003**	CMCC	*Staphylococcus aureus*	NP	NP
**CMCC 26112**	CMCC	*Staphylococcus aureus*	NP	NP
**ATCC 12600**	Phagelux AgriHealth	*Staphylococcus aureus*	r11:r19:r12:r12:r21:r10:r34:r24:r34:r22:r25	t1029
**ATCC 25923**	Hopebiol	*Staphylococcus aureus*	NP	NP
**ATCC 29213**	Hopebiol	*Staphylococcus aureus*	r26:r23:r17:r34:r17:r20:r17:r12:r17:r16	t002
**ATCC 6538**	Phagelux AgriHealth	*Staphylococcus aureus*	r14:r12:r21:r17:r34:r34:r34:r33:r34	t3297
**Newman**	Phagelux AgriHealth	*Staphylococcus aureus*	r11:r19:r12:r21:r17:r34:r24:r34:r22:r25	t008
**NRS384**	Phagelux AgriHealth	*Staphylococcus aureus*	r11:r19:r12:r21:r17:r34:r24:r34:r22:r25	t008
**RN4220**	Phagelux AgriHealth	*Staphylococcus aureus*	r11:r19:r12:r12:r21:r17:r34:r24:r34:r22:r25	t211
**StaPW08**	Phagelux AgriHealth	*Staphylococcus aureus*	r26:r16	t586
**StaPW09**	Phagelux AgriHealth	*Staphylococcus aureus*	r07:r06:r17:r21:r34:r34:r22:r34	t164
**StaPW10**	Phagelux AgriHealth	*Staphylococcus aureus*	r26:r16	t586
**StaPW100**	Phagelux AgriHealth	*Staphylococcus aureus*	r07:r21:r17:r13:r13:r34:r34:r34:r33:r13	t5348
**StaPW101**	Phagelux AgriHealth	*Staphylococcus aureus*	r07:r12:r21:r17:r13:r13:r13:r34:r34:r34:r34:r24:r13	*

**StaPW102**	Phagelux AgriHealth	*Staphylococcus aureus*	r07:r12:r21:r17:r13:r34:r34:r33:r13	t3155
**StaPW105**	Phagelux AgriHealth	*Staphylococcus aureus*	r04:r34:r21:r17:r21:r17:r34:r22:r25	t4549
**StaPW106**	Phagelux AgriHealth	*Staphylococcus aureus*	r04:r20:r17:r20:r17:r25	t3592
**StaPW107**	Phagelux AgriHealth	*Staphylococcus aureus*	r04:r20:r17:r20:r17:r25:r34	t437
**StaPW108**	Phagelux AgriHealth	*Staphylococcus aureus*	r04:r20:r17:r20:r17:r25:r34	t437
**StaPW112**	Phagelux AgriHealth	*Staphylococcus aureus*	r04:r20:r17:r20:r17:r25:r34	t437
**StaPW113**	Phagelux AgriHealth	*Staphylococcus aureus*	r04:r20:r17:r20:r17:r25:r34	t437
**StaPW118**	Phagelux AgriHealth	*Staphylococcus aureus*	r07:r23:r12:r21:r17:r34	t189
**StaPW119**	Phagelux AgriHealth	*Staphylococcus aureus*	r11:r10:r21:r17:r34:r24:r34:r22:r25:r25	t701
**StaPW12**	Phagelux AgriHealth	*Staphylococcus aureus*	r07:r23:r12:r21:r17:r34	t189
**StaPW122**	Phagelux AgriHealth	*Staphylococcus aureus*	r07:r23:r21:r17:r12:r23:r02:r12:r23	t796
**StaPW123**	Phagelux AgriHealth	*Staphylococcus aureus*	r17:r45:r16:r34	*
**StaPW125**	Phagelux AgriHealth	*Staphylococcus aureus*	r21:r17:r34:r24:r34:r17:r34:r24:r34:r22:r25	*
**StaPW126**	Phagelux AgriHealth	*Staphylococcus aureus*	r04:r20:r17:r45:r16:r34	t172
**StaPW127**	Phagelux AgriHealth	*Staphylococcus aureus*	r04:r20:r17:r20:r17:r25:r34	t437
**StaPW128**	Phagelux AgriHealth	*Staphylococcus aureus*	r04:r20:r17:r45:r639:r16:r34	*
**StaPW129**	Phagelux AgriHealth	*Staphylococcus aureus*	r04:r20:r17:r20:r17:r25:r34	t437
**StaPW13**	Phagelux AgriHealth	*Staphylococcus aureus*	r07:r23:r12:r21:r17:r34	t189
**StaPW132**	Phagelux AgriHealth	*Staphylococcus aureus*	r04:r20:r17:r25:r34	t441
**StaPW134**	Phagelux AgriHealth	*Staphylococcus aureus*	r07:r23:r21:r17:r34:r12:r23:r02:r12:r23	t091
**StaPW135**	Phagelux AgriHealth	*Staphylococcus aureus*	r07:r23:r21:r16:r34:r33:r13	t127
**StaPW136**	Phagelux AgriHealth	*Staphylococcus aureus*	r07:r06:r17:r21:r34:r34:r22:r34	t164
**StaPW14**	Phagelux AgriHealth	*Staphylococcus aureus*	r26:r16	t586
**StaPW141**	Phagelux AgriHealth	*Staphylococcus aureus*	r08:r16:r02:r25:r02:r25:r34:r25	t571
**StaPW142**	Phagelux AgriHealth	*Staphylococcus aureus*	r15:r12:r16:r02:r24:r24	t030
**StaPW143**	Phagelux AgriHealth	*Staphylococcus aureus*	r07:r23:r12:r23:r02:r12:r23	t547
**StaPW146**	Phagelux AgriHealth	*Staphylococcus aureus*	r15:r12:r16:r02:r24:r24	t030
**StaPW147**	Phagelux AgriHealth	*Staphylococcus aureus*	r04:r20:r17:r20:r17:r25:r34	t437
**StaPW148**	Phagelux AgriHealth	*Staphylococcus aureus*	r15:r12:r16:r02:r24:r24	t030
**StaPW149**	Phagelux AgriHealth	*Staphylococcus aureus*	r15:r12:r16:r02:r16:r02:r25:r17:r24	t021
**StaPW15**	Phagelux AgriHealth	*Staphylococcus aureus*	r26:r17:r34:r34:r17:r20:r17:r17:r17:r16	t2460
**StaPW150**	Phagelux AgriHealth	*Staphylococcus aureus*	r26:r23:r17:r34:r17:r20:r17:r12:r17:r16	t002
**StaPW151**	Phagelux AgriHealth	*Staphylococcus aureus*	r07:r23:r21:r17:r34:r12:r23:r02:r12:r23	t091
**StaPW152**	Phagelux AgriHealth	*Staphylococcus aureus*	r11:r10:r21:r17:r34:r24:r34:r22:r25:r25	t701
**StaPW158**	Phagelux AgriHealth	*Staphylococcus aureus*	r15:r12:r16:r16:r16:r16:r02:r25:r17	t007
**StaPW159**	Phagelux AgriHealth	*Staphylococcus aureus*	r26:r16	t586
**StaPW160**	Phagelux AgriHealth	*Staphylococcus aureus*	r14:r12:r21:r17:r34:r34:r34:r33:r34	t3297
**StaPW161**	Phagelux AgriHealth	*Staphylococcus aureus*	r56:r16:r02:r25:r17:r24	*
**StaPW164**	Phagelux AgriHealth	*Staphylococcus aureus*	r15:r12:r16:r02:r25:r17:r24	t037
**StaPW165**	Phagelux AgriHealth	*Staphylococcus aureus*	r15:r12:r16:r16:r02:r16:r02:r25:r17:r24	t318

**StaPW168**	Phagelux AgriHealth	*Staphylococcus aureus*	r15:r12:r16:r02:r24:r24	t030
**StaPW169**	Phagelux AgriHealth	*Staphylococcus aureus*	r15:r12:r16:r02:r24:r24	t030
**StaPW171**	Phagelux AgriHealth	*Staphylococcus aureus*	r08:r16:r02:r25:r02:r25:r34:r25	t571
**StaPW172**	Phagelux AgriHealth	*Staphylococcus aureus*	r08:r16:r02:r25:r02:r25:r34:r25	t571
**StaPW173**	Phagelux AgriHealth	*Staphylococcus aureus*	r07:r23:r21:r17:r12:r23:r02:r12:r23	t796
**StaPW174**	Phagelux AgriHealth	*Staphylococcus aureus*	r07:r23:r21:r17:r34:r12:r23:r02:r12:r23	t091
**StaPW18**	Phagelux AgriHealth	*Staphylococcus aureus*	r26:r23:r17:r34:r20:r17:r12:r12:r17:r16	t5576
**StaPW180**	Phagelux AgriHealth	*Staphylococcus aureus*	r15:r12:r16:r02:r25:r17:r24	t037
**StaPW181**	Phagelux AgriHealth	*Staphylococcus aureus*	r08:r16:r02:r25:r17:r24	t138
**StaPW182**	Phagelux AgriHealth	*Staphylococcus aureus*	r08:r16:r02:r25:r17:r24	t138
**StaPW183**	Phagelux AgriHealth	*Staphylococcus aureus*	r08:r16:r02:r25:r17:r24	t138
**StaPW184**	Phagelux AgriHealth	*Staphylococcus aureus*	r08:r16:r02:r25:r17:r24	t138
**StaPW185**	Phagelux AgriHealth	*Staphylococcus aureus*	r08:r16:r02:r25:r17:r24	t138
**StaPW187**	Phagelux AgriHealth	*Staphylococcus aureus*	r11:r10:r21:r17:r34:r24:r34:r22:r25:r25	t701
**StaPW189**	Phagelux AgriHealth	*Staphylococcus aureus*	r07:r23:r21:r17:r34:r12:r23:r02:r12:r23	t091
**StaPW19**	Phagelux AgriHealth	*Staphylococcus aureus*	r11:r10:r21:r17:r34:r24:r34:r22:r25:r25	t701
**StaPW190**	Phagelux AgriHealth	*Staphylococcus aureus*	r04:r20:r17:r20:r17:r25:r34	t437
**StaPW191**	Phagelux AgriHealth	*Staphylococcus aureus*	r15:r21:r16:r02:r25:r17:r24	t338
**StaPW192**	Phagelux AgriHealth	*Staphylococcus aureus*	r08:r34:r34:r12:r34:r12:r12:r23:r02:r12:r23	*
**StaPW193**	Phagelux AgriHealth	*Staphylococcus aureus*	r26:r23:r17:r34:r17:r20:r17:r12:r17:r16	t002
**StaPW194**	Phagelux AgriHealth	*Staphylococcus aureus*	r07:r23:r12:r21:r17:r34	t189
**StaPW195**	Phagelux AgriHealth	*Staphylococcus aureus*	r07:r23:r12:r21:r17:r34	t189
**StaPW196**	Phagelux AgriHealth	*Staphylococcus aureus*	r04:r20:r17:r20:r17:r25:r34	t437
**StaPW197**	Phagelux AgriHealth	*Staphylococcus aureus*	r26:r23:r17:r34:r17:r20:r17:r12:r17:r16	t002
**StaPW198**	Phagelux AgriHealth	*Staphylococcus aureus*	r04:r02:r12:r21:r17:r34:r22:r25	t377
**StaPW199**	Phagelux AgriHealth	*Staphylococcus aureus*	r07:r23:r21:r16:r34:r33:r13	t127
**StaPW200**	Phagelux AgriHealth	*Staphylococcus aureus*	r26:r23:r17:r34:r17:r20:r17:r12:r17:r16	t002
**StaPW201**	Phagelux AgriHealth	*Staphylococcus aureus*	r26:r23:r17:r34:r17:r20:r17:r12:r17:r16	t002
**StaPW202**	Phagelux AgriHealth	*Staphylococcus aureus*	r26:r23:r17:r12:r17:r16	t062
**StaPW203**	Phagelux AgriHealth	*Staphylococcus aureus*	r04:r25:r34	t3401
**StaPW204**	Phagelux AgriHealth	*Staphylococcus aureus*	r07:r23:r21:r17:r34:r12:r23:r02:r12:r23	t091
**StaPW205**	Phagelux AgriHealth	*Staphylococcus aureus*	r07:r23:r12:r21:r17:r34	t189
**StaPW206**	Phagelux AgriHealth	*Staphylococcus aureus*	r07:r23:r12:r21:r12:r17:r20:r17:r12:r12:r17	t148
**StaPW207**	Phagelux AgriHealth	*Staphylococcus aureus*	r04:r20:r17:r20:r17:r25:r34:r34	t3523
**StaPW208**	Phagelux AgriHealth	*Staphylococcus aureus*	r04:r02:r12:r21:r17:r34:r22:r25	t377
**StaPW209**	Phagelux AgriHealth	*Staphylococcus aureus*	r07:r23:r12:r21:r12:r17:r20:r17:r12:r12:r17	t148
**StaPW21**	Phagelux AgriHealth	*Staphylococcus aureus*	r15:r12:r16:r02:r24:r24	t030
**StaPW210**	Phagelux AgriHealth	*Staphylococcus aureus*	r04:r02:r12:r21:r17:r34:r22:r25	t377
**StaPW211**	Phagelux AgriHealth	*Staphylococcus aureus*	r07:r23:r21:r17:r12:r23:r02:r12:r23	t796
**StaPW212**	Phagelux AgriHealth	*Staphylococcus aureus*	r07:r23:r21:r17:r34:r12:r23:r02:r12:r23	t091

**StaPW213**	Phagelux AgriHealth	*Staphylococcus aureus*	r11:r10:r21:r17:r34:r24:r34:r22:r25:r25	t701
**StaPW220**	Phagelux AgriHealth	*Staphylococcus aureus*	r07:r23:r12:r21:r17:r34	t189
**StaPW221**	Phagelux AgriHealth	*Staphylococcus aureus*	r26:r23:r17:r34:r20:r17:r12:r17:r16	t311
**StaPW222**	Phagelux AgriHealth	*Staphylococcus aureus*	r26:r23:r17:r12:r17:r16	t062
**StaPW223**	Phagelux AgriHealth	*Staphylococcus aureus*	r07:r21:r17:r13:r13:r34:r34:r34:r33:r13	t5348
**StaPW224**	Phagelux AgriHealth	*Staphylococcus aureus*	r04:r34:r21:r17:r21:r17:r34:r22:r25	t4549
**StaPW225**	Phagelux AgriHealth	*Staphylococcus aureus*	r04:r34:r21:r17:r21:r17:r34:r22:r25	t4549
**StaPW226**	Phagelux AgriHealth	*Staphylococcus aureus*	r08:r16:r02:r25:r02:r25:r34:r34:r25	t5635
**StaPW227**	Phagelux AgriHealth	*Staphylococcus aureus*	r04:r34:r21:r17:r21:r17:r34:r22:r25	t4549
**StaPW228**	Phagelux AgriHealth	*Staphylococcus aureus*	r11:r12:r197:r17:r34:r24:r34:r22:r25	t9101
**StaPW229**	Phagelux AgriHealth	*Staphylococcus aureus*	r26:r23:r17:r34:r17:r20:r17:r12:r17:r16	t002
**StaPW230**	Phagelux AgriHealth	*Staphylococcus aureus*	r26:r23:r05:r17:r25:r17:r25:r16:r28	t309
**StaPW231**	Phagelux AgriHealth	*Staphylococcus aureus*	r07:r23:r12:r13:r17:r16:*	*
**StaPW232**	Phagelux AgriHealth	*Staphylococcus aureus*	r04:r20:r17:r20:r17:r25:r34	t437
**StaPW233**	Phagelux AgriHealth	*Staphylococcus aureus*	r07:r23:r21:r17:r34:r12:r23	t1943
**StaPW234**	Phagelux AgriHealth	*Staphylococcus aureus*	r15:r12:r16:r02:r16:r02:r25:r17:r24:r24:r24	t018
**StaPW235**	Phagelux AgriHealth	*Staphylococcus aureus*	r15:r12:r16:r02:r16:r02:r25:r17:r24:r24:r24	t018
**StaPW236**	Phagelux AgriHealth	*Staphylococcus aureus*	r11:r12:r197:r17:r34:r24:r34:r22:r25	t9101
**StaPW237**	Phagelux AgriHealth	*Staphylococcus aureus*	r04:r02:r12:r21:r17:r34:r22:r25	t377
**StaPW238**	Phagelux AgriHealth	*Staphylococcus aureus*	r11:r12:r197:r17:r34:r24:r34:r22:r25	t9101
**StaPW239**	Phagelux AgriHealth	*Staphylococcus aureus*	r04:r20:r17:r20:r17:r25:r34	t437
**StaPW24**	Phagelux AgriHealth	*Staphylococcus aureus*	r04:r20:r17:r20:r17:r25:r34	t437
**StaPW240**	Phagelux AgriHealth	*Staphylococcus aureus*	r04:r34:r21:r17:r21:r17:r34:r22:r25	t4549
**StaPW241**	Phagelux AgriHealth	*Staphylococcus aureus*	r26:r23:r17:r34:r20:r17:r12:r17:r16	t311
**StaPW242**	Phagelux AgriHealth	*Staphylococcus aureus*	r04:r02:r12:r34:r22:r25	t4047
**StaPW243**	Phagelux AgriHealth	*Staphylococcus aureus*	r08:r16:r02:r25	t1456
**StaPW244**	Phagelux AgriHealth	*Staphylococcus aureus*	r07:r23:r12:r21:r17:r34	t189
**StaPW245**	Phagelux AgriHealth	*Staphylococcus aureus*	r07:r23:r12:r21:r17:r34	t189
**StaPW247**	Phagelux AgriHealth	*Staphylococcus aureus*	r04:r20:r17:r20:r17:r25:r34	t437
**StaPW248**	Phagelux AgriHealth	*Staphylococcus aureus*	r26:r16:r28	t1977
**StaPW249**	Phagelux AgriHealth	*Staphylococcus aureus*	r11:r10:r21:r17:r34:r24:r34:r22:r25:r25	t701
**StaPW251**	Phagelux AgriHealth	*Staphylococcus aureus*	r04:r02:r12:r34:r22	*
**StaPW252**	Phagelux AgriHealth	*Staphylococcus aureus*	r26:r23:r23:r17:r34:r17:r20:r17:r12:r17	t2532
**StaPW253**	Phagelux AgriHealth	*Staphylococcus aureus*	r26:r17:r34:r17:r20:r17:r12:r16	t855
**StaPW255**	Phagelux AgriHealth	*Staphylococcus aureus*	r07:r23:r12:r21:r17:r34	t189
**StaPW256**	Phagelux AgriHealth	*Staphylococcus aureus*	r26:r23:r17:r02:r17:r12:r12:r17:r16	*
**StaPW257**	Phagelux AgriHealth	*Staphylococcus aureus*	r07:r23:r12:r21:r17:r34	t189
**StaPW26**	Phagelux AgriHealth	*Staphylococcus aureus*	r08:r16:r02:r25:r02:r25:r34:r25	t571
**StaPW260**	Phagelux AgriHealth	*Staphylococcus aureus*	r15:r12:r16:r02:r24:r24	t030
**StaPW261**	Phagelux AgriHealth	*Staphylococcus aureus*	r15:r12:r16:r02:r24:r24	t030

**StaPW262**	Phagelux AgriHealth	*Staphylococcus aureus*	r15:r12:r16:r02:r24:r24	t030
**StaPW263**	Phagelux AgriHealth	*Staphylococcus aureus*	r13:r12:r17:r17:r23:r18:r17	*
**StaPW264**	Phagelux AgriHealth	*Staphylococcus aureus*	r15:r12:r16:r02:r24:r24	t030
**StaPW265**	Phagelux AgriHealth	*Staphylococcus aureus*	r14:r12:r17:r17:r23:r18:r17	t2019
**StaPW266**	Phagelux AgriHealth	*Staphylococcus aureus*	r15:r12:r16:r02:r24:r24	t030
**StaPW267**	Phagelux AgriHealth	*Staphylococcus aureus*	r08:r16:r02:r25:r17:r24	t138
**StaPW269**	Phagelux AgriHealth	*Staphylococcus aureus*	r08:r16:r02:r25:r17:r24	t138
**StaPW27**	Phagelux AgriHealth	*Staphylococcus aureus*	r26:r17:r20:r17:r20:r17:r12:r16	t5353
**StaPW277**	Phagelux AgriHealth	*Staphylococcus aureus*	r13:r12:r17:r17:r17	*
**StaPW278**	Phagelux AgriHealth	*Staphylococcus aureus*	r07:r23:r21:r17:r34:r12:r23:r02:r12:r23	t091
**StaPW279**	Phagelux AgriHealth	*Staphylococcus aureus*	r15:r12:r16:r02:r24:r24	t030
**StaPW28**	Phagelux AgriHealth	*Staphylococcus aureus*	r04:r20:r17:r20:r17:r25:r34	t437
**StaPW282**	Phagelux AgriHealth	*Staphylococcus aureus*	r12:r17:r17:r23:r23:r18:r17:r17	*
**StaPW283**	Phagelux AgriHealth	*Staphylococcus aureus*	r11:r25:r25	t2915
**StaPW284**	Phagelux AgriHealth	*Staphylococcus aureus*	r07:r23:r21:r16:r34:r33:r13	t127
**StaPW285**	Phagelux AgriHealth	*Staphylococcus aureus*	r15:r12:r16:r02:r24:r24	t030
**StaPW286**	Phagelux AgriHealth	*Staphylococcus aureus*	r15:r12:r16:r02:r25:r17:r24	t037
**StaPW287**	Phagelux AgriHealth	*Staphylococcus aureus*	r15:r12:r16:r02:r25:r17:r24	t037
**StaPW288**	Phagelux AgriHealth	*Staphylococcus aureus*	r15:r12:r16:r02:r24:r24	t030
**StaPW289**	Phagelux AgriHealth	*Staphylococcus aureus*	r15:r12:r16:r02:r25:r17:r24	t037
**StaPW29**	Phagelux AgriHealth	*Staphylococcus aureus*	r07:r23:r21:r16:r34:r33:r13	t127
**StaPW292**	Phagelux AgriHealth	*Staphylococcus aureus*	r08:r16:r02:r25:r17:r24	t138
**StaPW293**	Phagelux AgriHealth	*Staphylococcus aureus*	r08:r16:r02:r25:r02:r25:r34:r25	t571
**StaPW294**	Phagelux AgriHealth	*Staphylococcus aureus*	r04:r20:r17:r20:r17:r25:r34	t437
**StaPW295**	Phagelux AgriHealth	*Staphylococcus aureus*	r15:r12:r16:r02:r25:r17:r24	t037
**StaPW296**	Phagelux AgriHealth	*Staphylococcus aureus*	r15:r12:r16:r02:r25:r17:r24	t037
**StaPW297**	Phagelux AgriHealth	*Staphylococcus aureus*	r08:r16:r02:r25:r17:r24	t138
**StaPW298**	Phagelux AgriHealth	*Staphylococcus aureus*	r08:r16:r02:r25:r17:r24	t138
**StaPW299**	Phagelux AgriHealth	*Staphylococcus aureus*	r08:r16:r02:r25:r17:r24	t138
**StaPW30**	Phagelux AgriHealth	*Staphylococcus aureus*	r08:r16:r02:r25:r02:r25:r34:r24:r25	t034
**StaPW301**	Phagelux AgriHealth	*Staphylococcus aureus*	r11:r19:r12:r21:r17:r34:r24:r34:r22:r25	t008
**StaPW302**	Phagelux AgriHealth	*Staphylococcus aureus*	r26:r23:r17:r34:r17:r20:r17:r12:r17:r16	t002
**StaPW303**	Phagelux AgriHealth	*Staphylococcus aureus*	r07:r23:r21:r16:r34:r33:r13	t127
**StaPW304**	Phagelux AgriHealth	*Staphylococcus aureus*	r15:r12:r16:r02:r24:r24	t030
**StaPW305**	Phagelux AgriHealth	*Staphylococcus aureus*	r26:r23:r17:r34:r17:r20:r17:r12:r17:r16	t002
**StaPW306**	Phagelux AgriHealth	*Staphylococcus aureus*	r11:r10:r21:r17:r34:r24:r34:r22:r25:r25	t701
**StaPW307**	Phagelux AgriHealth	*Staphylococcus aureus*	r13:r17:r17:r16:r23:r18:r17	*
**StaPW308**	Phagelux AgriHealth	*Staphylococcus aureus*	r14:r12:r17:r23:r18:r17	t2091
**StaPW309**	Phagelux AgriHealth	*Staphylococcus aureus*	r04:r20:r17:r20:r17:r25:r34	t437
**StaPW31**	Phagelux AgriHealth	*Staphylococcus aureus*	r04:r20:r17:r20:r17:r25:r34	t437

**StaPW310**	Phagelux AgriHealth	*Staphylococcus aureus*	r07:r23:r12:r21:r17:r34	t189
**StaPW311**	Phagelux AgriHealth	*Staphylococcus aureus*	r04:r02:r12:r21:r17:r34:r22:r25	t377
**StaPW312**	Phagelux AgriHealth	*Staphylococcus aureus*	r04:r20:r17:r20:r17:r25:r34	t437
**StaPW313**	Phagelux AgriHealth	*Staphylococcus aureus*	r04:r20:r17:r20:r17:r25:r34	t437
**StaPW314**	Phagelux AgriHealth	*Staphylococcus aureus*	r07:r23:r12:r21:r17:r34	t189
**StaPW315**	Phagelux AgriHealth	*Staphylococcus aureus*	r11:r10:r21:r17:r34:r24:r22:r25:r25:r25	*
**StaPW316**	Phagelux AgriHealth	*Staphylococcus aureus*	r07:r23:r21:r17:r34:r12:r23:r02:r12:r23	t091
**StaPW317**	Phagelux AgriHealth	*Staphylococcus aureus*	r04:r20:r17:r20:r17:r25:r34	t437
**StaPW318**	Phagelux AgriHealth	*Staphylococcus aureus*	r04:r20:r17:r20:r17:r25:r34	t437
**StaPW319**	Phagelux AgriHealth	*Staphylococcus aureus*	r11:r10:r21:r17:r34:r24:r34:r22:r25	t304
**StaPW32**	Phagelux AgriHealth	*Staphylococcus aureus*	r07:r23:r21:r17:r34:r12:r23:r02:r12:r23	t091
**StaPW320**	Phagelux AgriHealth	*Staphylococcus aureus*	r04:r20:r17:r20:r17:r25:r34	t437
**StaPW322**	Phagelux AgriHealth	*Staphylococcus aureus*	r26:r23:r05:r17:r25:r17:r25:r16:r28	t309
**StaPW323**	Phagelux AgriHealth	*Staphylococcus aureus*	r07:r21:r17:r34:r34:r34:r34:r33:r34	t4015
**StaPW324**	Phagelux AgriHealth	*Staphylococcus aureus*	r13:r13:r12:r17:r17	*
**StaPW325**	Phagelux AgriHealth	*Staphylococcus aureus*	r08:r16:r02:r25:r02:r25:r02:r25:r34:r24:r25	t2582
**StaPW326**	Phagelux AgriHealth	*Staphylococcus aureus*	r07:r12:r21:r17:r13:r34:r34:r34:r33:r13	t3622
**StaPW327**	Phagelux AgriHealth	*Staphylococcus aureus*	r07:r12:r21:r17:r13:r13:r34:r34:r33:r13	t1376
**StaPW328**	Phagelux AgriHealth	*Staphylococcus aureus*	r07:r23:r21:r16:r34:r33:r13	t127
**StaPW329**	Phagelux AgriHealth	*Staphylococcus aureus*	r07:r23:r12:r21:r17:r34	t189
**StaPW33**	Phagelux AgriHealth	*Staphylococcus aureus*	r11:r10:r21:r17:r34:r24:r34:r22:r25:r25	t701
**StaPW330**	Phagelux AgriHealth	*Staphylococcus aureus*	r04:r12:r21:r12:r41:r20:r17:r12:r12:r41	*
**StaPW331**	Phagelux AgriHealth	*Staphylococcus aureus*	r04:r02:r12:r21:r17:r34:r02:r25	t3386
**StaPW332**	Phagelux AgriHealth	*Staphylococcus aureus*	r04:r20:r17:r20:r17:r25:r34	t437
**StaPW333**	Phagelux AgriHealth	*Staphylococcus aureus*	r15:r12:r16:r02:r24:r24	t030
**StaPW334**	Phagelux AgriHealth	*Staphylococcus aureus*	r04:r02:r12:r21:r17:r34:r22:r25	t377
**StaPW335**	Phagelux AgriHealth	*Staphylococcus aureus*	r04:r20:r17:r20:r17:r25:r34	t437
**StaPW336**	Phagelux AgriHealth	*Staphylococcus aureus*	r11:r10:r21:r17:r34:r24:r34:r22:r25:r25	t701
**StaPW337**	Phagelux AgriHealth	*Staphylococcus aureus*	r07:r12:r21:r17:r13:r13:r34:r34:r33:r13	t1376
**StaPW338**	Phagelux AgriHealth	*Staphylococcus aureus*	r07:r23:r12:r21:r17:r34	t189
**StaPW339**	Phagelux AgriHealth	*Staphylococcus aureus*	r07:r23:r12:r21:r17:r34	t189
**StaPW34**	Phagelux AgriHealth	*Staphylococcus aureus*	r08:r02:r25:r02:r25:r34:r24:r25	t1928
**StaPW340**	Phagelux AgriHealth	*Staphylococcus aureus*	r08:r16:r02:r25:r02:r25:r34:r24:r25	t034
**StaPW341**	Phagelux AgriHealth	*Staphylococcus aureus*	r26:r23:r17:r34:r17:r20:r17:r12:r17:r16	t002
**StaPW342**	Phagelux AgriHealth	*Staphylococcus aureus*	r04:r02:r12:r21:r17:r34:r22:r25	t377
**StaPW343**	Phagelux AgriHealth	*Staphylococcus aureus*	r07:r23:r12:r21:r12:r17:r20:r17:r12:r12:r17	t148
**StaPW344**	Phagelux AgriHealth	*Staphylococcus aureus*	r26:r23:r17:r34:r17:r02:r17:r12:r17:r16	t1062
**StaPW346**	Phagelux AgriHealth	*Staphylococcus aureus*	r26:r17:r20:r17:r20:r17:r12:r16	t5353
**StaPW347**	Phagelux AgriHealth	*Staphylococcus aureus*	r07:r23:r12:r21:r17:r34	t189
**StaPW348**	Phagelux AgriHealth	*Staphylococcus aureus*	r26:r17:r20:r16	t1568

**StaPW349**	Phagelux AgriHealth	*Staphylococcus aureus*	r04:r20:r17:r20:r17:r25:r34	t437
**StaPW35**	Phagelux AgriHealth	*Staphylococcus aureus*	r04:r20:r17:r25:r34	t441
**StaPW350**	Phagelux AgriHealth	*Staphylococcus aureus*	r07:r06:r17:r21:r34:r34:r22:r34	t164
**StaPW351**	Phagelux AgriHealth	*Staphylococcus aureus*	r07:r23:r21:r17:r34:r12:r23:r02:r12:r23	t091
**StaPW352**	Phagelux AgriHealth	*Staphylococcus aureus*	r04:r34:r21:r17:r21:r17:r34:r22:r25	t4549
**StaPW353**	Phagelux AgriHealth	*Staphylococcus aureus*	r04:r20:r17:r20:r17:r25:r34	t437
**StaPW354**	Phagelux AgriHealth	*Staphylococcus aureus*	r15:r12:r16:r02:r16:r02:r25:r17:r24	t021
**StaPW355**	Phagelux AgriHealth	*Staphylococcus aureus*	r04:r20:r17:r20:r17:r25:r34	t437
**StaPW356**	Phagelux AgriHealth	*Staphylococcus aureus*	r08:r16:r02:r25:r02:r25:r34:r25	t571
**StaPW357**	Phagelux AgriHealth	*Staphylococcus aureus*	r15:r12:r16:r02:r24:r24	t030
**StaPW358**	Phagelux AgriHealth	*Staphylococcus aureus*	r15:r12:r16:r02:r24:r24	t030
**StaPW359**	Phagelux AgriHealth	*Staphylococcus aureus*	r15:r12:r16:r02:r24:r24	t030
**StaPW36**	Phagelux AgriHealth	*Staphylococcus aureus*	r04:r20:r17:r20:r17:r25:r34	t437
**StaPW360**	Phagelux AgriHealth	*Staphylococcus aureus*	r15:r12:r16:r02:r24:r24	t030
**StaPW361**	Phagelux AgriHealth	*Staphylococcus aureus*	r15:r12:r16:r02:r24:r24	t030
**StaPW362**	Phagelux AgriHealth	*Staphylococcus aureus*	r15:r12:r16:r02:r24:r24	t030
**StaPW363**	Phagelux AgriHealth	*Staphylococcus aureus*	r15:r12:r16:r02:r24:r24	t030
**StaPW366**	Phagelux AgriHealth	*Staphylococcus aureus*	r04:r20:r17:r45:r16:r34	t172
**StaPW367**	Phagelux AgriHealth	*Staphylococcus aureus*	r08:r16:r02:r25:r02:r25:r34:r25	t571
**StaPW38**	Phagelux AgriHealth	*Staphylococcus aureus*	r08:r16:r02:r25:r17:r24	t138
**StaPW39**	Phagelux AgriHealth	*Staphylococcus aureus*	r08:r16:r02:r25:r17:r24	t138
**StaPW40**	Phagelux AgriHealth	*Staphylococcus aureus*	r08:r16:r02:r25:r17:r24	t138
**StaPW41**	Phagelux AgriHealth	*Staphylococcus aureus*	r15:r12:r16:r02:r25:r17:r24	t037
**StaPW42**	Phagelux AgriHealth	*Staphylococcus aureus*	r04:r20:r17:r20:r17:r25:r34	t437
**StaPW43**	Phagelux AgriHealth	*Staphylococcus aureus*	r15:r12:r16:r02:r25:r17:r24	t037
**StaPW44**	Phagelux AgriHealth	*Staphylococcus aureus*	r15:r12:r16:r02:r25:r17:r24	t037
**StaPW45**	Phagelux AgriHealth	*Staphylococcus aureus*	r04:r20:r17:r20:r17:r25:r34	t437
**StaPW46**	Phagelux AgriHealth	*Staphylococcus aureus*	r08:r16:r02:r25:r17:r24	t138
**StaPW47**	Phagelux AgriHealth	*Staphylococcus aureus*	r15:r12:r16:r02:r24:r24	t030
**StaPW48**	Phagelux AgriHealth	*Staphylococcus aureus*	r15:r12:r16:r02:r24:r24	t030
**StaPW49**	Phagelux AgriHealth	*Staphylococcus aureus*	r15:r12:r16:r02:r24:r24	t030
**StaPW50**	Phagelux AgriHealth	*Staphylococcus aureus*	r04:r20:r17:r20:r17:r25:r34	t437
**StaPW51**	Phagelux AgriHealth	*Staphylococcus aureus*	r07:r23:r12:r34:r12:r12:r23:r02:r12:r23	t346
**StaPW52**	Phagelux AgriHealth	*Staphylococcus aureus*	r04:r02:r12:r21:r17:r34:r22:r25	t377
**StaPW53**	Phagelux AgriHealth	*Staphylococcus aureus*	r07:r23:r21:r17:r34:r12:r23:r02:r12:r23	t091
**StaPW54**	Phagelux AgriHealth	*Staphylococcus aureus*	r13:r12:r17:r17:r17	*
**StaPW56**	Phagelux AgriHealth	*Staphylococcus aureus*	r26:r23:r17:r12:r17:r16	t062
**StaPW58**	Phagelux AgriHealth	*Staphylococcus aureus*	r26:r23:r17:r34:r17:r20:r17:r12:r17:r16	t002
**StaPW59**	Phagelux AgriHealth	*Staphylococcus aureus*	r14:r12:r21:r17:r34:r34:r34:r33:r34	t3297
**StaPW60**	Phagelux AgriHealth	*Staphylococcus aureus*	r04:r20:r17:r20:r17:r25:r34	t437

**StaPW61**	Phagelux AgriHealth	*Staphylococcus aureus*	r15:r12:r16:r16:r16:r16:r02:r25:r17	t007
**StaPW62**	Phagelux AgriHealth	*Staphylococcus aureus*	r15:r12:r16:r02:r16:r02:r25:r17:r24	t021
**StaPW63**	Phagelux AgriHealth	*Staphylococcus aureus*	r26:r23:r17:r34:r17:r20:r17:r12:r17:r16	t002
**StaPW64**	Phagelux AgriHealth	*Staphylococcus aureus*	r07:r23:r21:r17:r34:r12:r23:r02:r12:r23	t091
**StaPW65**	Phagelux AgriHealth	*Staphylococcus aureus*	r11:r10:r21:r17:r34:r24:r34:r22:r25:r25	t701
**StaPW70**	Phagelux AgriHealth	*Staphylococcus aureus*	r15:r12:r16:r02:r24:r24	t030
**StaPW78**	Phagelux AgriHealth	*Staphylococcus aureus*	r13:r13:r12:r17:r17	*
**StaPW79**	Phagelux AgriHealth	*Staphylococcus aureus*	r08:r16:r02:r25:r02:r25:r34:r24:r24:r25	t1793
**StaPW80**	Phagelux AgriHealth	*Staphylococcus aureus*	r15:r12:r16:r02:r24:r24	t030
**StaPW81**	Phagelux AgriHealth	*Staphylococcus aureus*	r15:r12:r16:r02:r25:r17:r24	t037
**StaPW82**	Phagelux AgriHealth	*Staphylococcus aureus*	r15:r12:r16:r02:r25:r17:r24	t037
**StaPW83**	Phagelux AgriHealth	*Staphylococcus aureus*	r08:r16:r02:r25:r17:r24	t138
**StaPW84**	Phagelux AgriHealth	*Staphylococcus aureus*	r16:r02:r25:r17:r24	*
**StaPW85**	Phagelux AgriHealth	*Staphylococcus aureus*	r02:r25:r17:r24	*
**StaPW86**	Phagelux AgriHealth	*Staphylococcus aureus*	r04:r20:r17:r20:r17:r25:r34	t437
**StaPW87**	Phagelux AgriHealth	*Staphylococcus aureus*	r04:r12:r41:r13:r12:r12:r66	*
**StaPW88**	Phagelux AgriHealth	*Staphylococcus aureus*	r07:r23:r02:r12:r23	t803
**StaPW89**	Phagelux AgriHealth	*Staphylococcus aureus*	r07:r23:r12:r21:r17:r34	t189
**StaPW90**	Phagelux AgriHealth	*Staphylococcus aureus*	r04:r21:r12:r41:r20:r17:r12:r12:r17	t078
**StaPW91**	Phagelux AgriHealth	*Staphylococcus aureus*	r07:r23:r12:r21:r17:r34	t189
**StaPW92**	Phagelux AgriHealth	*Staphylococcus aureus*	r26:r23:r17:r20:r17:r12:r16	t6662
**StaPW93**	Phagelux AgriHealth	*Staphylococcus aureus*	r15:r12:r16:r02:r24:r24	t030
**StaPW94**	Phagelux AgriHealth	*Staphylococcus aureus*	r15:r12:r16:r02:r24:r24	t030
**StaPW121**	Phagelux AgriHealth	*Staphylococcus capitis*	NA	NA
**StaPW365**	Phagelux AgriHealth	*Staphylococcus capitis*	NA	NA
**StaPW96**	Phagelux AgriHealth	*Staphylococcus capitis*	NA	NA
**StaPW97**	Phagelux AgriHealth	*Staphylococcus capitis*	NA	NA
**StaPW133**	Phagelux AgriHealth	*Staphylococcus cohnii*	NA	NA
**CMCC 26069**	CMCC	*Staphylococcus epidermidis*	NA	NA
**StaPW95**	Phagelux AgriHealth	*Staphylococcus epidermidis*	NA	NA
**ATCC 35984**	Phagelux AgriHealth	*Staphylococcus epidermidis*	NA	NA
**StaPW109**	Phagelux AgriHealth	*Staphylococcus epidermidis*	NA	NA
**StaPW111**	Phagelux AgriHealth	*Staphylococcus epidermidis*	NA	NA
**StaPW116**	Phagelux AgriHealth	*Staphylococcus epidermidis*	NA	NA
**StaPW117**	Phagelux AgriHealth	*Staphylococcus epidermidis*	NA	NA
**StaPW120**	Phagelux AgriHealth	*Staphylococcus epidermidis*	NA	NA
**StaPW131**	Phagelux AgriHealth	*Staphylococcus epidermidis*	NA	NA
**StaPW137**	Phagelux AgriHealth	*Staphylococcus epidermidis*	NA	NA
**StaPW138**	Phagelux AgriHealth	*Staphylococcus epidermidis*	NA	NA
**StaPW139**	Phagelux AgriHealth	*Staphylococcus epidermidis*	NA	NA

**StaPW140**	Phagelux AgriHealth	*Staphylococcus epidermidis*	NA	NA
**StaPW153**	Phagelux AgriHealth	*Staphylococcus epidermidis*	NA	NA
**StaPW154**	Phagelux AgriHealth	*Staphylococcus epidermidis*	NA	NA
**StaPW155**	Phagelux AgriHealth	*Staphylococcus epidermidis*	NA	NA
**StaPW156**	Phagelux AgriHealth	*Staphylococcus epidermidis*	NA	NA
**StaPW157**	Phagelux AgriHealth	*Staphylococcus epidermidis*	NA	NA
**StaPW246**	Phagelux AgriHealth	*Staphylococcus epidermidis*	NA	NA
**StaPW73**	Phagelux AgriHealth	*Staphylococcus epidermidis*	NA	NA
**StaPW98**	Phagelux AgriHealth	*Staphylococcus epidermidis*	NA	NA
**StaPW104**	Phagelux AgriHealth	*Staphylococcus haemolyticus*	NA	NA
**StaPW110**	Phagelux AgriHealth	*Staphylococcus haemolyticus*	NA	NA
**StaPW115**	Phagelux AgriHealth	*Staphylococcus haemolyticus*	NA	NA
**StaPW74**	Phagelux AgriHealth	*Staphylococcus haemolyticus*	NA	NA
**StaPW124**	Phagelux AgriHealth	*Staphylococcus hominis*	NA	NA
**StaPW72**	Phagelux AgriHealth	*Staphylococcus hominis*	NA	NA
**StaPW76**	Phagelux AgriHealth	*Staphylococcus lugdunensis*	NA	NA
**ATCC 49444**	Mingzhou	*staphylococcus pseudintermedius*	NA	NA
**StaPW75**	Phagelux AgriHealth	*Staphylococcus saprophyticus*	NA	NA
**StaPW07**	Phagelux AgriHealth	CoNS isolate	NA	NA
**StaPW11**	Phagelux AgriHealth	CoNS isolate	NA	NA
**StaPW23**	Phagelux AgriHealth	CoNS isolate	NA	NA
**StaPW25**	Phagelux AgriHealth	CoNS isolate	NA	NA
**StaPW57**	Phagelux AgriHealth	CoNS isolate	NA	NA

TABLE S3 – Other Bacterial Strains

**Strain**	**Source**	**Organism name**
DH5α	Takara	*Escherichia coli*
BL21(DE3)	Sigma-Aldrich	*Escherichia coli*
CMCC 44113	CMCC	*Escherichia coli*
CMCC 50335	CMCC	*Salmonella enteritidis*
CMCC 50001	CMCC	*Salmonella paratyphi A*
CMCC 50094	CMCC	*Salmonella paratyphi B*
CMCC 50115	CMCC	*Salmonella typhimurium*
CMCC 52204	CMCC	*Yersinia enterocolitica*
CMCC 49005	CMCC	*Proteus mirabilis*
CMCC 10211	CMCC	*Pseudomonas aeruginosa*
CMCC 10104	CMCC	*Pesudomonas pyocyaneum*

CMCC 25001	CMCC	*Acinetobacter baumannii*
CMCC 63301	CMCC	*Bacillus cereus*
CMCC 63303	CMCC	*Bacillus cereus*
CMCC 63202	CMCC	*Bacillus pumilus*
CMCC 63501	CMCC	*Bacillus subtilis*
CMCC 28001	CMCC	*Micrococcus luteus*
CICC 24106	CICC	*Escherichia coli*
CICC 10389	CICC	*Escherichia coli*
CICC 21519	CICC	*Klebsiella pneumoniae*
CICC 22933	CICC	*Acinetobacter baumannii*
PacPW22	Phagelux AgriHealth	*Cutibacterium acnes*
PacPW7	Phagelux AgriHealth	*Cutibacterium acnes*
PacPW10	Phagelux AgriHealth	*Cutibacterium acnes*
BacPW31	Phagelux AgriHealth	*Streptococcus dysgalactiae*
BacPW01	Phagelux AgriHealth	*Streptococcus dysgalactiae*
BacPW10	Phagelux AgriHealth	*Streptococcus agalactiae*

TABLES S2 and S3 strain source information: Takara (Kusatsu, Shiga, Japan); Sigma-Aldrich (St. Louis, MO, USA); ATCC, American Type Culture Collection (Manassas, VA, USA); BNCC, BeNa Culture Collection (Henan, China); CMCC, National Center for Medical Culture Collections (Beijing, China); CICC, China Center of Industrial Culture Collection (China); Hopebiol (Qingdao, China); Mingzhou (Ningbo, China); Phagelux AgriHealth (Nanjing, China). Other abbreviations: NA, not available (i.e., no band was observed in a PCR); NP, not performed (for certain strains from qualified strain repositories).

SUPPLEMENTAL MOVIES

MOVIE S1 Rapid lysis of *S. aureus* by LYSG101 visualized by video microscopy. Log-phase *S. aureus* ATCC 29213 cells were washed and attached to a poly-L-lysine-coated glass-bottomed plate. The plate was washed, supplemented with DPBS, and placed on a microscope stage. Video

was captured continuously. A brief shift in the light plane is noticeable at the beginning of the movie, caused by the addition of LYSG101. Close-up video clips of specific cells are presented at the end of the movie.

MOVIE S2 Real-time visualization of bacterial clearing in liquid. A suspension of washed log-phase *S. aureus* CMCC 26003 in CATBS at an OD_{600} of 1.0 was divided into two T-25 Corning flasks (30 mL each). LYSG101 was added to one of the flasks to a final concentration of 50 µg/mL, and turbidity reduction was captured by video. A similar volume of vehicle was added to the control flask prior to imaging.

REFERENCES

1. Shopsin B, Gomez M, Montgomery SO, Smith DH, Waddington M, Dodge DE, Bost DA, Riehman M, Naidich S, Kreiswirth BN. 1999. Evaluation of protein A gene polymorphic region DNA sequencing for typing of *Staphylococcus aureus* strains. J Clin Microbiol 37:3556-63.
2. CLSI. 2021. Performance standards for antimicrobial susceptibility testing. 31st ed. CLSI supplement M100. Clinical and Laboratory Standards Institute, Wayne, PA.
3. CLSI. 2015. Methods for dilution antimicrobial susceptibility tests for bacteria that grow aerobically; approved standard — tenth edition, CLSI document M07-A10. Clinical and Laboratory Standards Institute, Wayne, PA.
4. Wei HY, H.; Yu, J. 2018. *Staphylococcus* lysin and use thereof. United States.
5. Yang H, Zhang H, Wang J, Yu J, Wei H. 2017. A novel chimeric lysin with robust antibacterial activity against planktonic and biofilm methicillin-resistant *Staphylococcus aureus*. Sci Rep 7:40182.
6. Gilmer DB, Schmitz JE, Euler CW, Fischetti VA. 2013. Novel bacteriophage lysin with broad lytic activity protects against mixed infection by *Streptococcus pyogenes* and methicillin-resistant *Staphylococcus aureus*. Antimicrob Agents Chemother 57:2743-50.
7. Schuch R, Lee HM, Schneider BC, Sauve KL, Law C, Khan BK, Rotolo JA, Horiuchi Y, Couto DE, Raz A, Fischetti VA, Huang DB, Nowinski RC, Wittekind M. 2014. Combination therapy with lysin CF-301 and antibiotic is superior to antibiotic alone for treating methicillin-resistant *Staphylococcus aureus*-induced murine bacteremia. J Infect Dis 209:1469-78.

8. Studier FW. 2005. Protein production by auto-induction in high density shaking cultures. Protein Expr Purif 41:207-34.
9. Moody J. 2010. Synergism Testing: Broth Microdilution Checkerboard and Broth Macrodilution Methods. *In* Garcia LS (ed), Clinical Microbiology Procedures Handbook, 3rd ed, vol 2. ASM Press, Washington, DC.
